# Understanding of catalytic ROS generation from defect-rich graphene quantum-dots for therapeutic effects in tumor microenvironment

**DOI:** 10.1186/s12951-021-01053-6

**Published:** 2021-10-26

**Authors:** Xichu Wang, Chuangang Hu, Zi Gu, Liming Dai

**Affiliations:** Australian Carbon Materials Centre (A-CMC), School of Chemical Engineering, University of New South Wales, Sydney, NSW 2052 Australia

**Keywords:** Carbon-based metal-free catalyst, Graphene quantum dot, Defect, Reactive oxygen species, Tumor microenvironment

## Abstract

Owing to their low cost, high catalytic efficiency and biocompatibility, carbon-based metal-free catalysts (C-MFCs) have attracted intense interest for various applications, ranging from energy through environmental to biomedical technologies. While considerable effort and progress have been made in mechanistic understanding of C-MFCs for non-biomedical applications, their catalytic mechanism for therapeutic effects has rarely been investigated. In this study, defect-rich graphene quantum dots (GQDs) were developed as C-MFCs for efficient ROS generation, specifically in the H_2_O_2_-rich tumor microenvironment to cause multi-level damages of subcellular components (even in nuclei). While a desirable anti-cancer performance was achieved, the catalytic performance was found to strongly depend on the defect density. It is for the first time that the defect-induced catalytic generation of ROS by C-MFCs in the tumor microenvironment was demonstrated and the associated catalytic mechanism was elucidated. This work opens a new avenue for the development of safe and efficient catalytic nanomedicine.

**Supplementary Information:**

The online version contains supplementary material available at 10.1186/s12951-021-01053-6.

## Introduction

Reactive Oxygen species (ROS) play an important role in maintaining biological functions. Cells inherently produce ROS that serves as cell signaling molecules for normal biological processes [1]. On the other hand, excessive ROS could induce oxidative stress to damage lipids, proteins and DNA, leading to apoptosis [1–4], necroptosis [2], and autophagic cell death [3, 5]. Thus, ROS-induced cell death could be one of the attractive options for cancer therapy if the cancer cell-specific ROS generation can be achieved. Nevertheless, the tumor microenvironment (TME) is a unique protective ecosystem that helps cancer cells to thrive [6], and a high level of hydrogen peroxide (H_2_O_2_) in TME has been demonstrated to promote tumor growth through apoptosis resistance, DNA alteration, and cell proliferation angiogenesis [7]. In the presence of an appropriate catalyst, hydrogen peroxide can be used for the catalytic generation of ROS, which, if excessively generated in a TME, could be used to kill cancer cells. Therefore, the catalyst is a key for ROS-induced cancer cell death.

By making redox reaction catalysts respond to specific biological milieu to achieve therapeutic effects, catalytic nanomaterials hold great promise to harness redox reactions for biochemical applications. In this context, catalytic nanomaterials have been used to mediate reactions to convert non-therapeutic compounds in the TME to therapeutic agents for specifically killing tumor cells. Recently, nanoparticles such as black phoshoruse nanosheet [8] and nanodots [9] were proposed to selectively induce ROS mediated cell death. In particular, iron nanoparticles [10–12] were used to disproportionate H_2_O_2_ in the TME to cytotoxic hydroxyl radicals (·OH) via Fenton(-like) reactions. Other nanoparticles, containing Mn, Co, Cu, or Ag element [6, 13–16], have also been demonstrated to act as nanozyme agents for inhibiting tumor growth. However, metal-containing nanoparticles with a strong chelation ability could interfere with the functionalities of biological molecules and/or tissues to inevitably expose biosafety risks [5]. By contrast, metal-free carbon-based catalysts (C-MFCs) are biocompatible, cost-effective, and multifunctional [17].

Since the discovery of nitrogen (N)-doped carbon nanotube catalyst as the first C-MFC for electrocatalytic oxygen reduction reaction (ORR) in 2009 [18], C-MFCs have been widely explored as efficient, low-cost alternatives to metal-based catalysts for energy, environmental and biomedical applications [19]. Of particular interest, N-doped carbon nanospheres have recently been investigated as nanozymes for catalytic cancer treatments by producing ·OH from H_2_O_2_ [20]_._ In this particular case, N-doping was attributed to being responsible for the generation of hydroxyl radicals. N-doping has been previously revealed to cause charge redistribution around adjacent carbon atoms to induce the catalytic activities [21]. Similarly, defects in carbon nanomaterials could also alter charge distributions to induce catalytic performance [22, 23], which has been confirmed by recent experimental and theoretical studies for energy-related reactions (e.g., ORR) [21–24]. So far, however, there is no study on the mechanistic understanding of defect-induced biocatalytic behaviors.

Owing to their small size, unique physicochemical properties, and biocatalytic behaviors, graphene quantum dots (GQDs) have been investigated as promising C-MFC-based therapeutic agents [25]. Recent studies demonstrated that graphene quantum dots functionalized with and without targeting agent (s) can cause DNA damage via ROS generation to suppress the growth of cancer cells [26, 27]. However, underlying mechanisms for the GQD-induced ROS generation have not yet been understood, though the associated ROS-induced DNA damage and biochemical anti-cancer effect have been widely studied. In this work, we have for the first time demonstrated the defect-induced ROS generation from GQD C-MFCs and hydrogen peroxide in TME and carried out the associated mechanistic study.

**Fig. 1:**
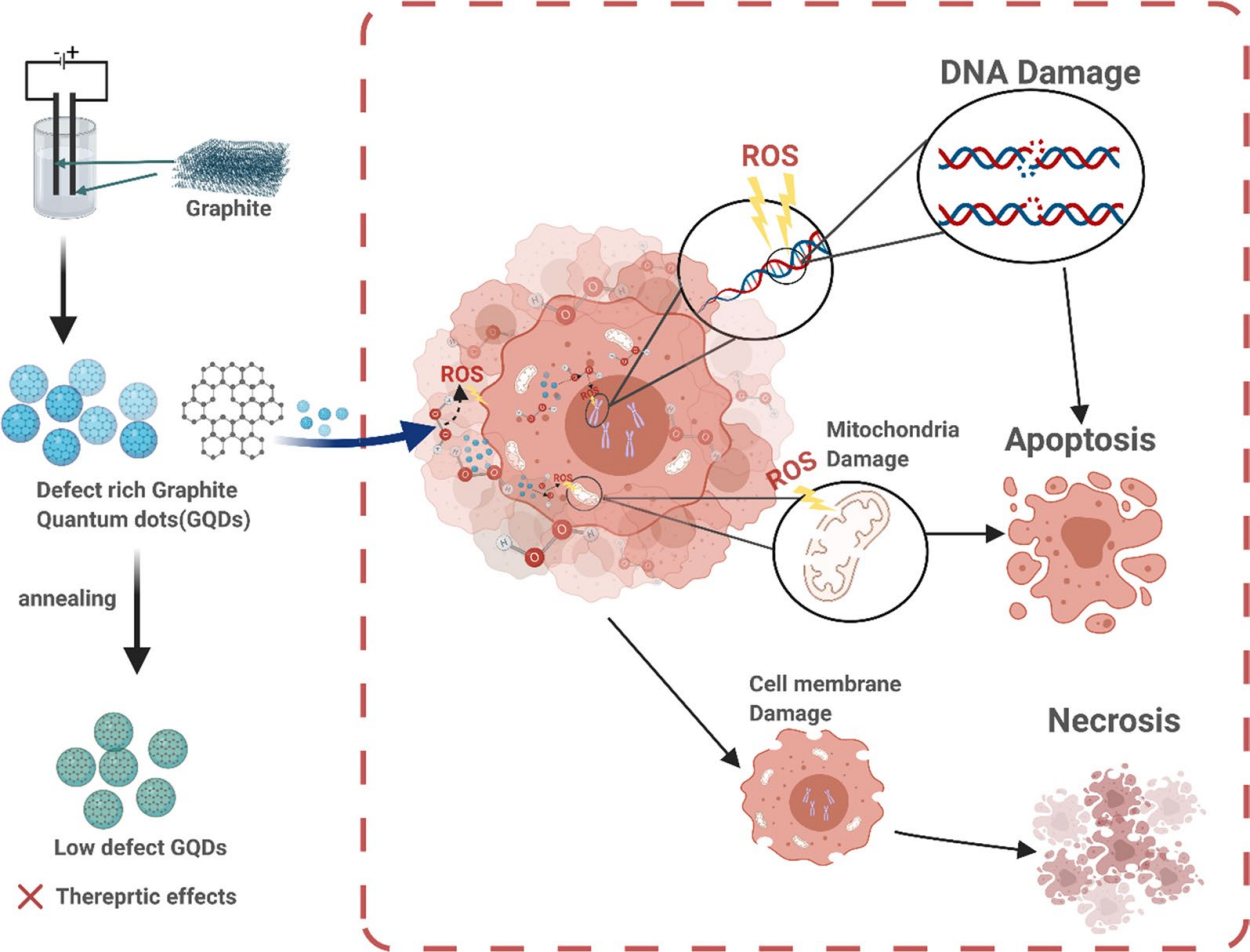
Schematic illustration of synthetic procedure and multi-target therapeutic mechanism of graphene quantum dots (GQDs). Figure created with BioRender

Herein, metal-free, doping-free, defect-rich GQDs were used to study the defect-induced catalytic generation of hydroxyl radicals (·OH) and the subsequent biological effects. Spin-trapped electron paramagnetic resonance (EPR) spectroscopy and colorimetric assay studies revealed that the catalytic performance strongly depends on the defect density. As schematically shown in Fig. 1, defect-rich GQDs could catalyze the efficient generation of hydroxyl radicals from hydrogen peroxide, especially in TME, to induce an efficacious anti-cancer effect even without the addition of any chemotherapeutic drugs. Our cellular and subcellular studies revealed multi-level anti-cancer mechanisms involving the cell membrane, mitochondria, and DNA damages by the defect-rich GQDs (Fig. 1). This newly observed GQD defect-induced catalytic ROS generation, along with the associated therapeutic effects in response to TME, represents the first proof of concept for the defect-induced metal-free catalytic therapeutic strategy towards safe and efficacious cancer treatment.

## Results and discussion

### Synthesis and structural characterization of GQDs

**Fig. 2:**
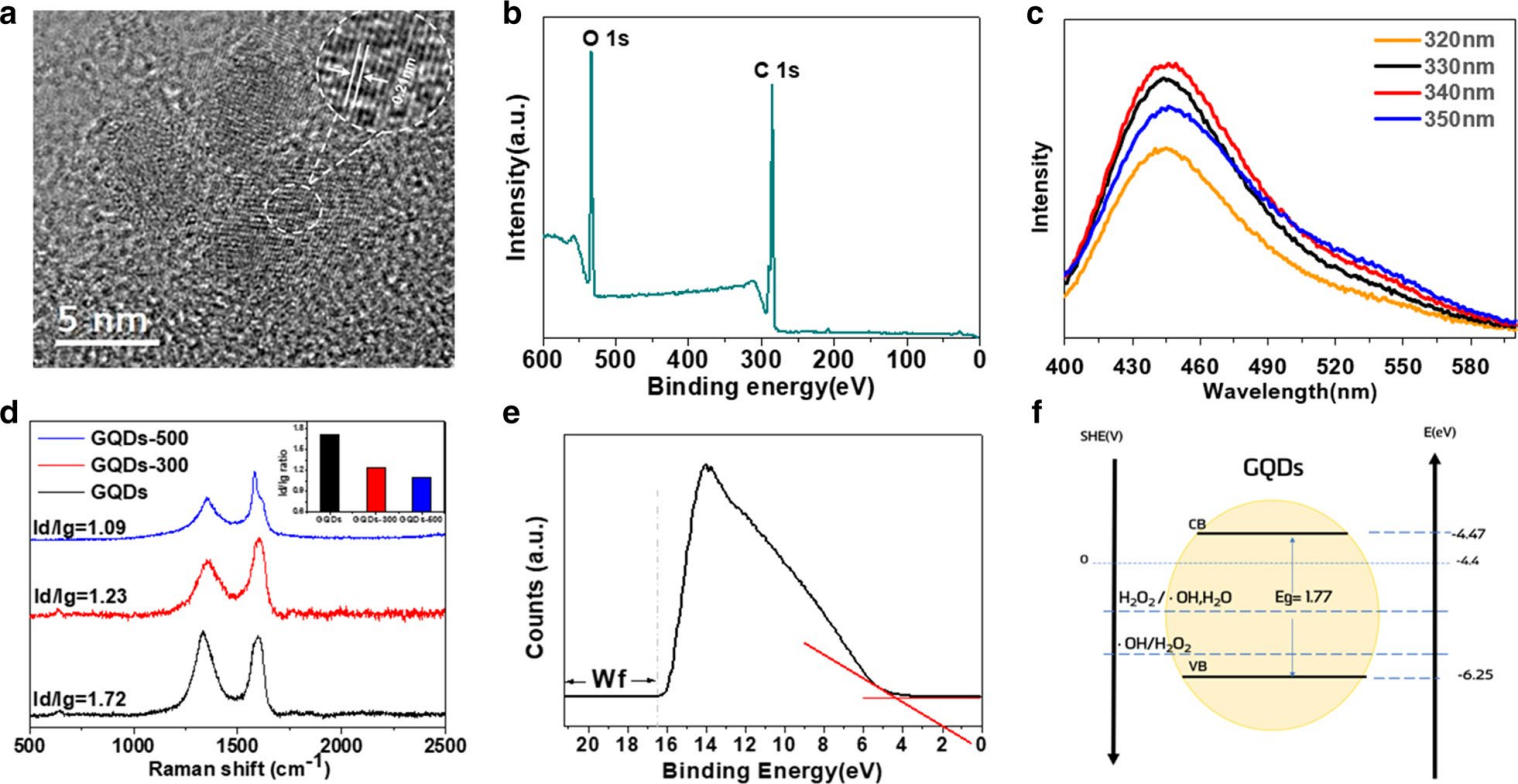
Physicochemical properties of GQDs. **a** TEM image (scale bar = 5 nm). **b** XPS survey spectra (insert: HR-XPS spectra of C1s). **c** Fluorescence emission spectrum at the excitation wavelength of different wavelengths ranging from 320 to 350 nm. **d** Raman spectra of GQDs with different defect densities (insert: Id/Ig ratio of GQDs with different defect densities). **e** UPS spectra of GQDs. **f** Band structure of GQDs. Conduction band(CV) = Valence band(VB) + ${\mathrm{E}}_{\mathrm{g}}^{\mathrm{opt}}$ [28]

The GQDs used in this study were synthesized and purified via a typical electrochemical method without further modification, according to a published procedure [29]. GQDs-300 and GQDs-500 were obtained by annealing the purified GQDs at 300 and 500 °C, respectively. The high-resolution transmission electron microscopic (HRTEM) examination revealed a spherical morphology with an average diameter of about 3.5 nm and crystal lattice spacing of 0.22 nm for the GQDs (Fig. 2a). The aqueous dispersion of GQDs was relatively stable at room temperature, which showed an average hydrodynamic particle size of 9.8 nm and zeta potential of − 28.5 mV without obivious change over 30 days (Additional file 1: Figs. S1 and S2).

To investigate the chemical compositions of the GQDs, we performed X-ray photoelectron spectroscopy (XPS) and Fourier-transform infrared spectroscopy (FT-IR). XPS analyses indicate the presence of C (72 at%) and O (26 at%) in GQDs (Fig. 2b). The corresponding ultraviolet–visible (UV–vis) absorption spectrum of the aqueous dispersion of GQDs shows a typical broad absorption peak at 230 nm attributable to $\pi ={\pi }^{*}$ transition of the aromatic C = O bond [30] (Additional file 1: Fig.S4). Figure 2c shows photoluminescent emissions at different excitation wavelengths (from 350 to 800 nm) for an aqueous dispersion of GQDs, indicating the highest emission peak at 450 nm under excitation at 340 nm (Fig. 2c). Figure 2d reproduces the Raman spectrum for the GQDs, which shows the presence of the crystalline G band (1596 cm^−1^) and disordered D band (1331 cm^−1^) with an I_D_/I_G_ ratio of 1.72, indicating a defect-rich structure [31].

For comparison, GQDs with relatively low defect densities were also synthesized via annealing under an argon atmosphere at 300 °C (GQDs-300) and 500 °C (GQDs-500), as mentioned above. As expected, annealing at higher temperatures could effectively reduce the defect density, as demonstrated by the decreased I_D_/I_G_ ratios in the order of 1.72, 1.23 and 1.09 for the pristine GQDs, GQDs-300, and GQDs-500, respectively (Fig. 2d).

Figure 2f shows the band structure diagram for the GQDs, which was obtained from the corresponding optical energy gap, deduced from the longest absorption edge (${\lambda }_{onset}, {\mathrm{E}}_{\mathrm{g}}^{\mathrm{opt}}= 1.77 eV)$ [32], and the highest occupied molecular orbital (HOMO) at the valence band from UPS (6.25 eV, Fig. 2e). The lowest unoccupied molecular orbital (LUMO) at the conduction band was then calculated to be: ${\mathrm{E}}_{\mathrm{g}}^{\mathrm{opt}}$ − HOMO = 1.77 − 6.25 = − 4.47 (Fig. 2f, Additional file 1: Table S1). Based on the calculated band structure and redox potentials shown in Fig. 2f, the GQDs-defect catalyzed hydroxyl radical generation from H_2_O_2_ is plausible.

### Defect-induced catalytic activity

**Fig. 3:**
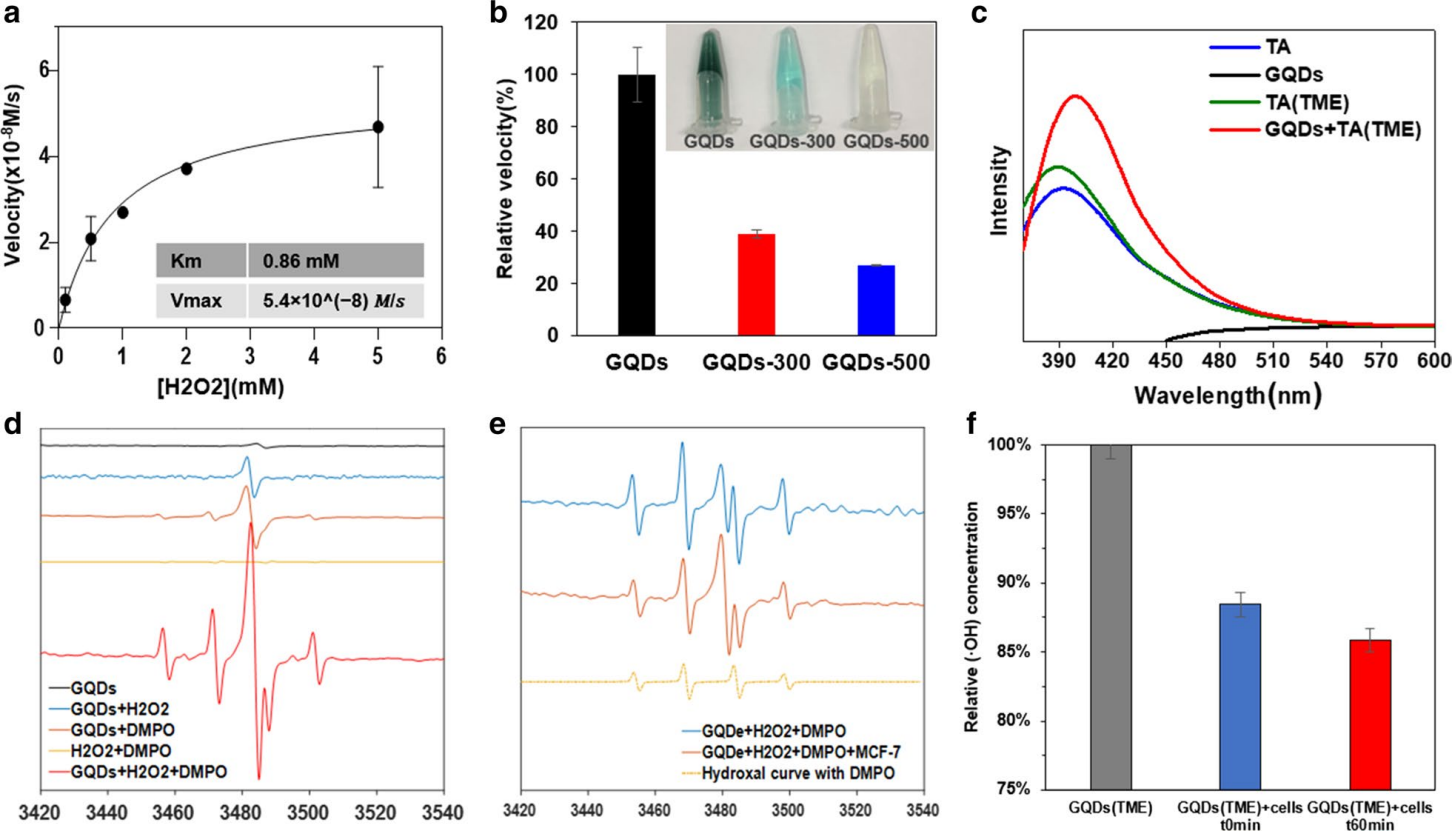
Catalytic performance of GQDs. **a** Michaelis–Menten kinetic profile. **b** Catalytic performance comparison via relative reaction velocity from TMB assay (in Hac/NaAc buffer) for GQDs (100 µg/ml) with different treatments (untreated, GQDs-300, GQDs-500) under TME (100 µM H_2_O_2_), insert: photo of TMB colorimetric assay at 10 min post-treatment of GQDs, GQDs-300, GQDs-500. **c** Fluorescence spectra of TA, GQDs, TA (TME) and GQDs + TA (TME) in PBS. The concentration of TA, TME (H_2_O_2_) and GQDs in PBS were 1 mM, 100 µM and 100 µg/ml, respectively. Hydroxyl radical identification via EPR using DMPO (1 mM) as the spin trap of: **d** GQDs (50 µg/ml) with and without H_2_O_2_ (1 mM), and **e** GQDs + H_2_O_2_ with and without adding MCF-7 cells (1 × 10^6^/ml). **f** Relative hydroxyl radical concentrations calculated from the signals in using Xenon quantify EPR software

We found that defect-rich GQDs possess a peroxidase-like activity that decomposes H_2_O_2_ into ·OH. To test the catalytic performance of the GQDs, we used the 3,3,5,5-tetramethylbenzidine (TMB) assay, which can be oxidized by ·OH from H_2_O_2_ decomposition to a blue product with absorbance at 652 nm [33]. As shown in Additional file 1: Figs. S6 and S7, GQDs significantly increased the reaction rates of H_2_O_2_ decomposition, followed by ·OH induced TMB oxidation. By plotting initial velocities against H_2_O_2_ concentrations, we performed the kinetic analysis and calculated the Michaelis–Menten constant (Km) and maximum initial velocity (Vmax) [20] to be 0.86 mM and 5.4 × 10^–8^ M/s, respectively (Fig. 3a and Additional file 1: Figs. S5 and S7), indicating the high catalytic activity of GQDs [10]. We further used TMB and GQDs of different defect densities to investigate the effect of the defect density on the catalytic activity. As shown in Fig. 3b, the reaction velocity decreased dramatically from 100% for GQDs to 39% for GQDs-300 and 26% for GQDs-500, indicating that a high defect density is critical to the enhanced catalytic activity. Experimental results revealed that the defective structure of GQDs induced the formation of positively and negatively charged carbon atoms to strengthen the chemisorption of hydrogen peroxide, leading to the reaction activation. This is because the defect structure breaks the perfect hexagon ring of graphitic carbon quantum dots, generating the unsymmetrical structure and altering the electron distribution of carbon skeleton [21, 34, 35].

To gain a mechanistic understanding of catalytic ROS generation, we used terephthalic acid (TA) as a specific hydroxyl radical probe to capture hydroxyl radicals for the generation of 2-hydroxyl terephthalic acid (TAOH) with fluorescence emission at 400 nm [36, 37]. The fluorescent intensity from a mixed solution of GQDs, H_2_O_2_ and TA significantly increased in comparison to the control solutions of TA, H_2_O_2_ + TA, and GQDs + TA, signifying the generation of abundant ·OH radicals from the interaction between GQDs and H_2_O_2_ (Fig. 3c and Additional file 1: Figs S8 and S9). This result suggests that GQDs possess the catalytic ability toward H_2_O_2_ decomposition to generate ·OH. The catalytic nature of GQDs was further supported by the same chemical structure before and after the reaction, demonstrated by FT-IR spectra of GQDs (Additional file 1: Fig. S3).

### In vitro ROS regulation and the associated biological effects in TME

As can be seen from the above discussion, the defect-rich GQDs can induce peroxidase-like catalytic reactions to decompose H_2_O_2_ into ·OH. To further confirm this, we firstly used electron paramagnetic resonance (EPR) spectroscopy, in conjunction with 5,5-dimethyl-1-pyrroline N-oxide (DMPO) as a typical nitrone spin trap, to identify ·OH species [38]. As shown in Fig. 3d, EPR spectra reveal a characteristic 1:2:2:1 signal pattern of ·OH in the sample containing both H_2_O_2_ and GQDs, but not in the samples without GQDs or H_2_O_2_. These results confirm that the GQDs can catalyze H_2_O_2_ decomposition to generate ·OH (Fig. 3d and Additional file 1: Fig.S10). To study the effect of hydroxyl radicals on cultured cancer cells, we incubated 1 × 10^6^/ml human breast cancer MCF-7 cells in a mixture of H_2_O_2_ and GQDs (50 µg/ml) for 60 min. Aliquots collected from the fresh mixture and after 10 min or 60 min were evaluated by EPR using the DMPO nitrone spin trap. As shown in Fig. 3e, f, MCF-7 cancer cells continuously consumed ·OH generated from the GQDs catalyzed H_2_O_2_ decomposition.

**Fig. 4:**
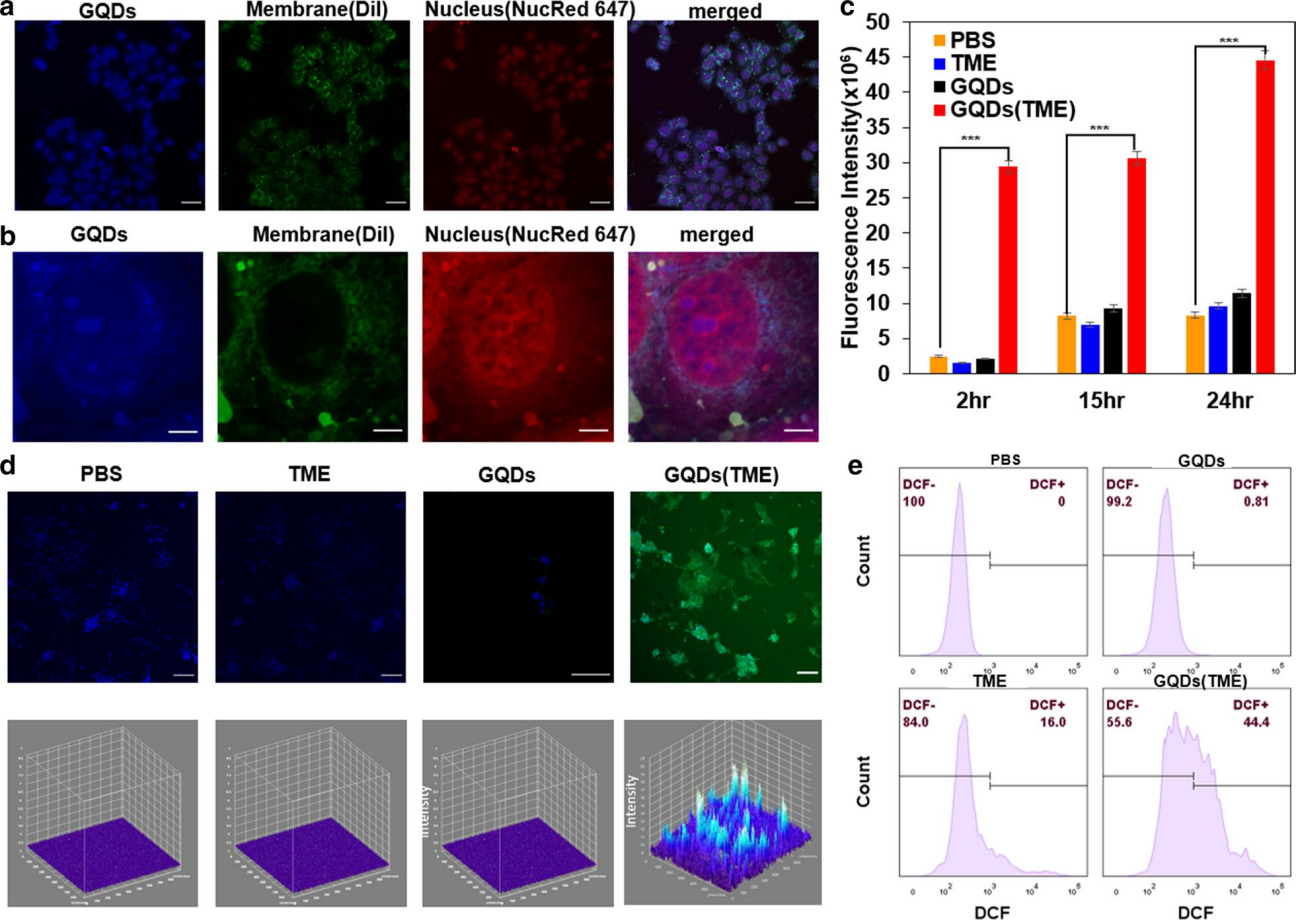
Cellular localization of GQDs and intracellular ROS generation. a, b Confocal microscopy images of GQDs-treated MCF-7 cells (**a:** scale bar: 50 µM, **b**:scale bar:10 µM). **c** Fluorescence intensity of DCF in 4T1 cell lines at 2, 15 and 24 h post-treatment of PBS, GQDs (100 µg/ml), TME (100 µM H_2_O_2_), GQD (TME) (100 µg/ml GQDs + 100 µM H_2_O_2_). **d** intracellular ROS level in 4T1 cells (green signals from DCF indicate ROS; blue signals from DAPI indicate nuclei), scale bar: 100 µm. **e** Quantified intracellular ROS level in 4T1 cells treated with agents: PBS, GQDs (100 µg/ml), TME (100 µM H_2_O_2_), GQD (TME) (100 µg/ml GQDs + 100 µM H_2_O_2_) for 2 h, measured by flow cytometry. Data are presented as mean ± standard deviation, and analyzed by unpaired Student’s two-tailed t-test, compared to PBS group. *p < 0.05, **p < 0.01, ***p < 0.001

To investigate the subcellular accumulation of GQDs, we performed the intracellular mapping of GQDs in MCF-7 cell culture by using dyes Dil and NucRed647 for visualizing plasma membrane and nuclei, respectively. As shown in confocal microscopy images given in Fig. 4a, b (Additional file 4: Movie S3 and Additional file 5: Movie S4), the GQDs with blue fluorescence emission under 405-nm excitation were found in both cytoplasm and nuclei in an aggregated form, and this finding is also further supported by TEM images in Additional file 1: Fig. S15. The driving force for GQDs to penetrate the cells and accumulate in nuclei is probably arising from both the size effect and the π–π stacking interactions between GQDs and DNA chains [26].

Hydrogen peroxide overexpression is a well-known hallmark of cancers, which can show a tenfold increase compared to normal cells [7]. Thus, the above-demonstrated catalytic capability of GQDs can be exploited to decompose H_2_O_2_ into cytotoxic ·OH radicals to efficiently kill cancer cells, particularly in the tumor microenvironment. In this study, we used DCFH-DA as a fluorescence probe to evaluate the intracellular radical generation in response to GQDs in the tumor microenvironment. It is known that DCFH-DA can permeate into cells and be intracellularly hydrolyzed to DCFH, then further oxidized by intracellular ROS to fluorescent product dichlorofluorescein (DCF) [39]. The time-dependent, TME-responsive, GQDs-induced ROS generation was demonstrated by DCF fluorescence intensities recorded on a photoluminescent spectrometer at 2, 15, and 24 h after the addition of GQDs. Figure 4c shows a burst production of ROS within the first 2-h treatment of GQDs in the tumor microenvironment, followed by a continued increase in the intracellular ROS level over 24 h. In contrast, a retarded low-level ROS generation was observed in control experiments without GQDs or TME (Fig. 4c). The intracellular ROS levels were also visualized by confocal microscopy (Fig. 4d). As expected, significant fluorescence emissions from DCF were observed in the treatment group of GQDs in TME, rather than in the control groups (i.e. PBS, TME, GQDs). The intracellular level of ROS was further quantified via flow cytometry, which demonstrated 0.81%, 16% and 44.4% of fluorescent cells for the GQDs, TME, and GQDs (TME), respectively (Fig. 4e), confirming, once again, that the defect-rich GQDs can catalyze the generation of a considerable amount of ROS in the tumor microenvironment.

**Fig. 5:**
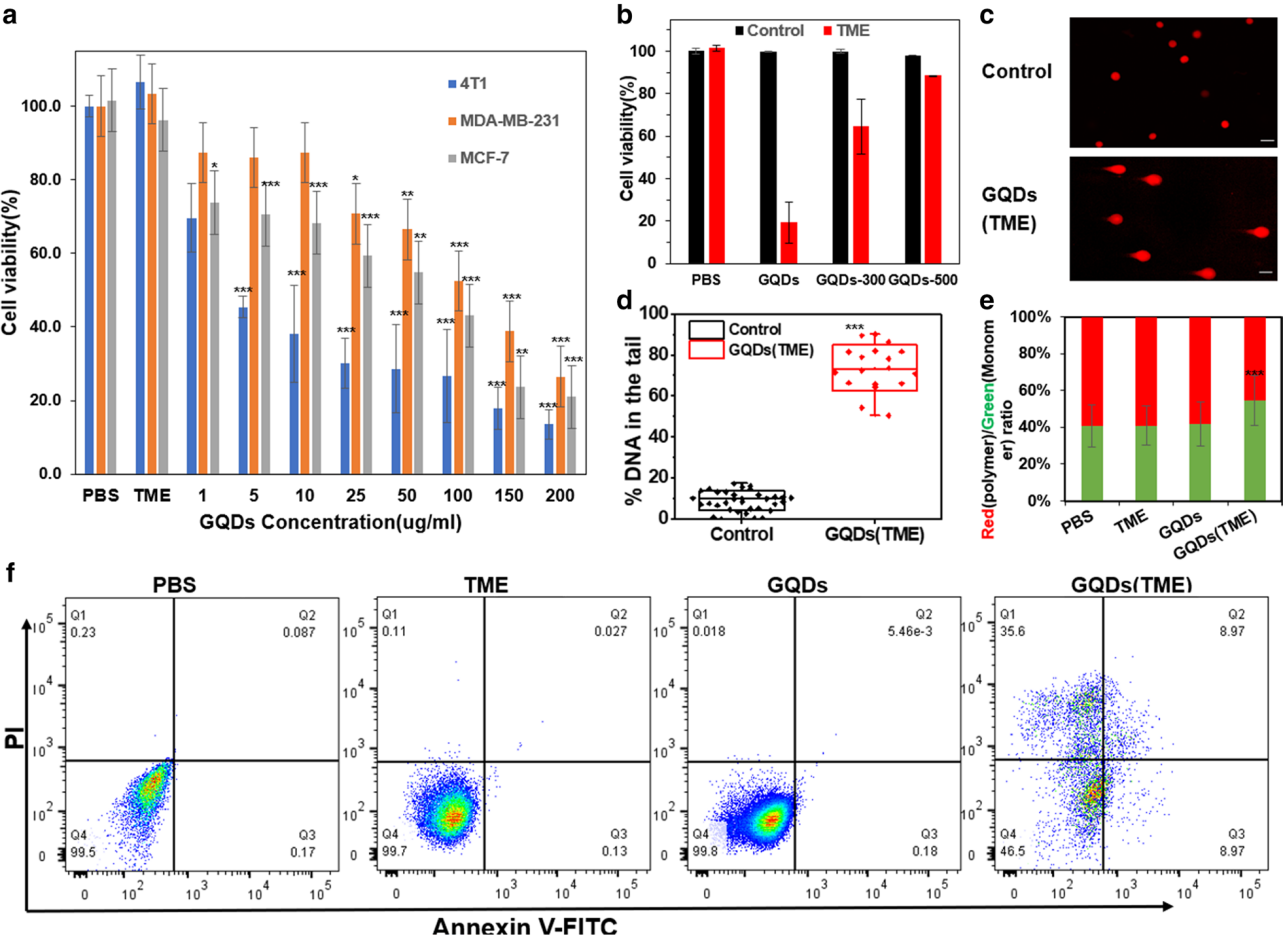
Therapeutic effects of GQD under TME. **a** Cell viability of 4T1/MDA-MB-231/MCF-7 cells treated with PBS, 100 µM H_2_O_2_ (TME), or GQDs (1–200 µg/ml) + 100 µM H_2_O_2_ under TME, for 24 h. **b** Cell viability of 4T1 cells treated with PBS, GQDs, GQDs-300 or GQDs-500 for 24 h. **c** Neutral comet assay of GQDs (200 µg/ml) under TME, scale bar: 200 µm. **d** Quantitative analysis of alkaline comet assay based on 30 cell samples. **e** The ratio of red (JC-1 polymer) over green (JC-1 monomer) fluorescence intensity of different treatment groups including PBS, TME, 100 µg/ml GQDs, and GQDs (1–100 µg/ml) + 100 µM H_2_O_2_, representing the changes in mitochondria membrane potential. **f** Apoptosis analysis via flow cytometry. Data are presented as mean ± SD. Statistical significance was analyzed by unpaired Student’s two-tailed t-test compared to the PBS group. *p < 0.05, **p < 0.01, ***p < 0.001

To evaluate the biologic effects of GQDs-mediated catalytic ROS generation, we measured the cell viability of breast cancer cells (4T1, MDA-MB-231, MCF-7) via CCK-8 assay by incubating three types of cell lines with GQDs at various concentrations under the TME-mimic conditions (100 µM H_2_O_2_) [40]. It is interesting to note that the addition of low-concentration hydrogen peroxide (≤ 100 µM) caused a slight increase in the cell viability of 4T1 (Additional file 1: Fig. S11), attributable to the fact that hydrogen peroxide could activate cancer cell growth by promoting cell malignant transformation and expression of H_2_O_2_-detoxifying compounds [7]. As shown in Fig. 5a, a GQDs concentration-specific and TME-selective cell viability was observed under TME conditions. Specifically, in 4T1 cell cultures, GQDs under TME achieved 80% inhibition on cell viability at 200 µg/ml GQDs, with a half maximum inhibitory concentration (IC50) of 50 µg/ml (Fig. 5a). When the GQD concentration increased from 1 to 200 µg/ml, cell viability decreased from 70% to 20%. Similarly, defect-rich GQDs exhibited dosage-dependent cytotoxicity on MDA-MB-231 and MCF-7 cells under the TME conditions. In contrast, there was no obvious change in cell viability for the control groups (i.e., PBS, TME, GQDs), and normal fibrolast cell NIH-3T3 can tolerate GQDs at the concentrations up to 250 µg/ml (Additional file 1: Fig. S12). The TME-responsive therapeutic effects of GQDs were further visualized by staining cells with propidium iodide (PI that stains dead cells) via time-lapse live-cell fluorescence imaging (Additional file 2: Movie S1 and Additional file 3: Movie S2). After the treatment with 100 µg/ml GQDs and 100 µM H_2_O_2_, the number of 4T1 cells did not increase while the cancer cells in control group (PBS) grew remarkably over 24 h. Meanwhile, the red fluorescence signals of non-viable cells (PI dye) significantly increased during the 24-h treatment period, in comparison to the control groups. These results indicate that GQDs can efficiently interact with cancer cells and suppressed cell growth in the H_2_O_2_-rich tumor microenvironment. Moreover, the anti-cancer efficiency of GQDs increased with increasing in the defect density of GQDs (Fig. 5b), consisting well with their defect-density-dependent catalytic activities (vide supra).

As a consequence, excessive ROS damages lipids, proteins, and DNA, leading to cell death [41]. To further investigate the anti-cancer mechanism of ROS generated from the GQDs-catalyzed H_2_O_2_ decomposition, we conducted comet assays to assess DNA damage. The round nuclear DNA without tails observed in the control groups indicates no obvious DNA damage, while the cells treated with GQDs under TME exhibited comet tails characteristic of DNA damage (Fig. 5c, d and Additional file 1: Fig. S14). Double-strand DNA breaks were revealed by a neutral comet assay (Fig. 5c). An average of 73% overall DNA damage was observed by alkaline comet assay (Fig. 5d). Therefore, the observed cell death could be attributed to the DNA damage-induced apoptosis caused by GQDs-catalyzed ROS generation. Mitochondria damage was also observed by measuring the mitochondrial membrane potential using lipophilic cationic dye JC-1 [42]. Specifically, JC-1 staining illustrated that mitochondrial potential was reduced after the GQDs treatment under TME (Fig. 5e), indicating significant mitochondria damage, which could also trigger the intrinsic apoptosis pathway [2, 43]. However, it cannot be ruled out that necrosis could also be triggered by the plasma membrane, a double layer of lipid and proteins, damage by the robust ROS level.

### Cell death pathway

To investigate the cell death pathways, we employed annexin V-FITC/PI and flow cytometry to examine the effect of GQDs on cell necrosis and apoptosis. FITC conjugated Annexin V is typically used to detect apoptosis via binding to phosphatidylserine (PS), which is an apoptosis marker that transports from the inner to the outer leaflet of the plasma membrane upon receiving pro-apoptotic signals [44, 45]. PI acts as an impermeant cell dye that binds to DNA and stains cells with the broken cell membrane to indicate necrosis or late-stage apoptosis [45, 46]. As shown in Fig. 5f, GQDs therapeutic treatment in TME elicited remarkable necrosis (25.6%, Q1, PI + /Annexin V-) and significant apoptosis (17.8%, Q2 + Q4, the sum of PI-/Annexin V + and PI + /Annexin V +) in 4T1 cells, compared to the controls (i.e. GQDs, TME) that showed neglectable necrosis and apoptosis. Notably, necrotic cell death is closely associated with cell membrane rupture that is likely caused by the peroxidase-like activity of GQDs and ·OH-induced lipid peroxidation. Apoptosis could be ascribed to mitochondria damage and/or DNA damage, which is consistent with the ROS cancer inhibition mechanism discussed above.

In summary, we have demonstrated the ROS generation from H_2_O_2_ decomposition catalyzed by defect-rich metal-free GQDs and elucidated the associated anticancer mechanism. Defect-induced, GQDs-concentration-dependent, tumor microenvironment-selective cancer cell apoptosis was observed. Specifically, defect-rich GQDs exhibited high catalytic activities for the generation of abundant hydroxyl radicals from H_2_O_2_ decomposition in the tumor microenvironment, showing remarkable inhibition effects toward cancer cells through multiple cellular component failures. The produced hydroxyl radicals caused DNA damage, interfered with mitochondria, and triggered subsequent apoptotic cell death pathways, while they could also damage cell membrane, leading to cell necrosis. The demonstrated proof-of-concept and mechanistic understanding of the ROS generation from H_2_O_2_ decomposition catalyzed by defect-rich GQDs in the tumor microenvironment for cancer cell apoptosis/necrosis via DNA damage/cell membrane damage provide a promising strategy for the design and development of carbon catalyst-based safe and efficacious cancer therapy. Owing to their facile synthesis, high selectivity to the tumor microenvironment, and highly efficient therapeutic ROS generation, we envision that the defect-rich GQDs hold great potential for high-performance nanomedicine in future preclinical and clinical trials.

## Methods

### Materials

Graphite rod (> 99.99999%), terephthalic acid (TA),5,5-dimethyl-1-pyrroline-N-oxide (DMPO), 3,5,5-tetramethylbenzidine (TMB), hydrogen peroxide, cell count kit-8 (CCK-8), were purchased from Sigma. Dulbecco's Modified Eagle Medium (DMEM, #11995073), Gibco Roswell Park Memorial Institute- 1640 (RMPI 1640, #22400105) medium were purchased from Thermofisher. Mili-Q water was used for synthesis and purification. Cell lines *4T1* (ATCCRL2541) and *NIH 3T3* (#93061524) were obtained from American Type Culture Collection (ATCC) and CellBank Australia, respectively; *MCF-7* and *MDA-MB-231* cells were obtained from Sigma.

### Synthesis of GQDs

GQDs were synthesized according to the published electrochemical method [29]**.** Briefly, two graphite rods (> 99.99999%, Sigma) as anode and cathode were inserted in ultrapure MiliQ water and subjected to 30 V from a direct current power supply. After the electrochemical reaction for 120 h, a dark brown solution was obtained, from which GQDs were collected and purified via centrifugation at 10000 rpm for 1 h, followed by filtration and freeze-drying.

### Synthesis of GQDs-300 and GQDs-500

To produce GQDs-300 and GQDs-500, GQDs were treated at 300 °C and 500 °C, respectively, for 30 min in the tube furnace with a ramp rate of 5 °C/min under argon atmosphere.

### Characterization of GQDs

HRTEM images were recorded on a JEOL-F200 scanning transmission electron microscope (S/TEM) equipped with a cold field emission gun and an EDS detector at an acceleration voltage of 200 kV. Raman spectra were recorded on a Rennishaw InVia 2 Raman Microscope (532 nm excitation, 1% laser power). X-ray photoelectron spectroscopy (XPS) was performed on a Thermo scientific ESCALAB250Xi spectrometer using a monochromatic Al K alpha X-ray source (energy 1486.68 eV) operated at 120 W (13.8 kV × 8.7 mA). Fourier-transform infrared spectroscopy was measured by Bruker compact FT-IR spectrometer ALPHA. Fluorescent emission spectroscopy was recorded on SHIMADZU RF-5301PC Spectrofluorophotometer. Ultraviolet photoelectron spectroscopy (UPS) data were obtained using the same instrument as XPS with a helium I ultraviolet source (energy 21.2 eV). The hydrodynamic size distribution (DLS) and zeta potential were measured on Zetasizer Nanoseries (Malvern).

### Catalytic performance evaluation

*Michaelis–Menten kinetics.* 3,3’,5,5’-Tetramethylbezine (TMB) was introduced to exam the catalytic performance and study the kinetic profiles, as the TMB undergoes a color oxidation reaction from colorless to blue to show absorption at 652 nm, which was measured by UV–vis (JASCO V-770). The TMB concentration was calculated from absorbance via the Beer-Lambert law [47]:

1 $$A=\varepsilon bc$$

where *A* is the absorbance, $\varepsilon$ is the molar absorption coefficient of the oxidized TMB [48] (39,000 M^−1^ cm^−1^), *b* is the optical length (1 cm), and *c* is the concentration of the oxidized TMB. Therefore, *c* was calculated by:

2 $$dc=\frac{dA}{\varepsilon b}=\frac{dA}{39000\times 1}(M)$$

The following condition was applied for relative activity test: TMB concentration: 800 µg/ml, Sample: 50 µL, H_2_O_2_ concentration: 1 mM, at room temperature. Michaelis–Menten equation was selected as the model to analyze the enzyme kinetic [49].

3 $${V}_{0}={V}_{max}\frac{[S]}{[S]\times {K}_{m}}$$

The Michaelis-Menton constant (Km) and the maximum velocity (Vm) were achieved with the use of curve-fitting programs GraphPad Prism 9 software (the Michaelis- Menton curve fitting) [33, 48].

#### Free radical identification

EPR signal was recorded on Bruker EMX X-Band EPR Spectrometer. In a typical experiment, DMPO (5 mM) [50] was added into the PBS buffer solution containing 50 µg/ml GQDs, to which 1 mM of H_2_O_2_ was added right before the measurements. The quantified analysis was performed using Xenon quantify EPR for peaks from 3450–3505 with the 2^nd^ baseline correction [51]. Radical concentrations were derived from the standard radical concentration curves, which were created based on the measurements of the ammonium iron sulfate Fe(NH_4_)_2_(SO_4_)_2_·6H_2_O and hydrogen peroxide [51, 52].

### Cell culture

Breast cancer cell lines 4T1, MCF-7, MDA-MB-231, and fibroblast cell line NIH-3T3 were cultured in complete growth media (CGM) at 37 °C with 5% CO_2_. CGM for MCF-7, MDA-MB-231, and NIH-3T3 cell lines are DMEM containing 10% fetal bovine serum (FBS) and 1% penicillin–streptomycin, i.e., penicillin 100 units/ml and streptomycin 100 µg/ml. CGM for the 4T1 cell line is RMPI-1640 containing 10% fetal bovine serum (FBS), and 1% penicillin–streptomycin. The cells were cultured at 37 °C in a humidified atmosphere with 5% CO_2_ and passaged when reaching ATCC recommended confluency.

### Cellular localization of GQDs

The cellular uptake biodistribution of GQDs was visualized by confocal microscopy. Briefly, coverslips were pre-disinfected and placed in a 24-well plate, followed by seeding 500 µl of MCF-7 cells in coverslip (1 × 10^5^ cells/ml). At 50% confluency, the CGM was removed, and cells were treated with PBS (control), TME (H_2_O_2_ 100 µM), and GQDs (100 µg/ml) only and GQDs (TME, H_2_O_2_ 100 µM) at 37 °C. After 24 h, the medium was removed, followed by rinsing with PBS three times. Cells were then stained with cell labels according to the manufacturer’s instructions. Five µL Dil cell membrane dye (Invitrogen™, V22885) and 995 µL serum-free medium were added into each well, incubated for 10 min at 37 °C. Dil membrane dye was then removed and cells were washed with PBS three times. Two drops of NucRed live 647 in 1 mL of fresh medium were added to each well. After 15 min incubation at 37 °C, cells were fixed with 4% PFA and mounted with prolong diamond Antifade mountant and observed under the confocal microscope (Zeiss 780/900).

### Intracellular ROS detection

Cell-permeable probe 2,7-Dichlorofluorescin diacetate (H_2_DCFDA) was used to analyze the intracellular ROS generation via plate reader, confocal microscopy and flow cytometry. Briefly, 500 µl of 4T1 cells were seeded in a 24-well plate (1 × 10^5^ cells/ml). At 50% confluency, cells were washed with PBS three times and then incubated with DCFH-DA (10 µM) in serum-free RPMI 1640 for 30 min at 37 °C in the dark. Then the medium was removed, followed by rinsing with PBS three times. Cells were treated with PBS (control), TME (H_2_O_2_ 100 µM) and GQDs (100 µg/ml) only and GQDs (TME, H_2_O_2_ 100 µM). At 2, 15, 24 h post-treatment, the DCF intensity was recorded on the plate reader (Molecular Device SpectraMax iD5).

To visualize the intracellular ROS level, 500 µl of 4T1 cells were seeded on the coverslip (1 × 10^5^ cells/ml). At 50% confluency, cells were washed with PBS three times then incubated with DCFH-DA (10 µM) in serum-free RPMI 1640 for 30 min at 37 °C in the dark. Then the medium was removed, followed by rinsing with PBS three times. Cells were treated with PBS (control), TME (H_2_O_2_ 100 µM) or GQDs (100 µg/ml) only, and GQDs (TME, H_2_O_2_ 100 µM) for 2-h incubation. Then, the cells were fixed with 4% PFA and mounted with Prolong diamond Antifade mountant for examination by confocal microscopy (Zeiss 780/900).

For flow cytometry analysis, 2 ml of 4T1 cells (3 × 10^5^ cells/ml) were seed into a 6-well plate. By following the same treatment procedure in the previous two experiments, cells were detached and collected, then measured immediately by flow cytometry (BD LSRFortessa) ($\lambda$ excitation = 488 nm and $\lambda$ emission = 530 nm). The time course intensity measurements were carried out on a plate reader Molecular Device SpectraMax iD5, and the same seeding and treatments were applied to 4T1. After different periods, the plate was measured under excitation and emission at 488 nm and 530 nm, respectively.

### Cell viability assay

CCK-8 assay was conducted to determine the in vitro cytotoxicity of GQDs. MCF-7/4T1/MDA-MB-231/3T3 cells were pre-incubated in a 96-well plate with a density of 5000 cells/well and cultured with 100 µl complete growth medium for 24 h. Different concentrations of GQDs (0–1000 µg/ml)/H_2_O_2_ (0 to 1000 µM$\mu$) were added and then incubated for 24 h. After that, 10 µl of CCk-8 reagent was added to each well and incubated for 4 h at 37 °C. The absorbance at 450 nm was measured using Molecular Device SpectraMax iD5. Data were presented as mean ± SD from three independent experiments.

### Apoptosis analysis

Cell Apoptosis was examined by annexin V/PI assay using Annexin V-FTIC apoptosis detection Kit (BNS500FI/100,eBioscience). 4T1 cells were pre-incubated in a 6-well plate at a density of 5000 cells/well and cultured with 2 ml of complete growth medium. At 50% confluency, cells were treated with PBS (control), TME (H_2_O_2_ 100 µM), and GQDs (100 µg/ml) only, and GQDs (TME, H_2_O_2_ 100 µM). After 24 h incubation, cells were detached and collected using trypsin–EDTA (centrifugation at 190 g for 5 min). Cells were washed with PBS twice and binding buffer once (centrifugation at 200 *g* for 3 min), and then resuspended in binding buffer containing Annexin V-FTIC (5:200) and incubated for 10 min at room temperature. Cells were washed with binding buffer and stain with PI (10:200) in binding buffer. The flow cytometry (BD LSRFortessa) was used to measure the fluorescence of Annexin V‐FITC and PI. All flow cytometry data were analyzed using Flowjo v10.

### DNA damage Study

#### Comet assay

4T1/MCF-7/MDA-MB-231 cells were seeded in 6-well plates (1 × 10^5^ cells/ml). At 50% confluency, complete growth media were replaced with the medium containing different treatment agents (i.e. PBS, TME (H_2_O_2_ 100 µM) or GQDs (100 µg/ml) only, and GQDs (TME, H_2_O_2_ 100 µM). After 24-h treatment, cells were detached with trypsin and resuspended in ice-cold PBS (1 × 10^5^ cells/ml), followed by cell lysis at 4 °C in dark for 2 h. Comet assay was then performed according to the protocol from the manufacturer. Briefly, 1% agarose gel was made by adding 1 g of agarose into 100 ml MiliQ water using the microwave method, and the gel was placed in a 37 °C water bath for at least 20 min to cool. 1 × 10^5^/ml cell samples were mixed with agarose (at 37 °C) at a ratio of 1:10 (v/v) and 50 μl mixture was immediately pipetted onto Comet Slides. Slides were placed at 4 °C in the dark for 30 min. The slides were immersed in lysis Solution for 60 min. Excess buffer was drained from slides which were then immersed in electrophoresis buffer (TBE for Neutral Comet and AES for Alkaline Comet) for 20 min. After that, the horizontal electrophoresis cell (Bio-Rad) was filled with electrophoresis buffers. Electrophoresis was conducted at 30 V for 30 min in dark. Slides were gently removed from the electrophoresis tray and rinsed twice with Milli Q water and 70% ethanol [53, 54]. Slides were dried and stained with SYBR Safe according to the manufacturer’s instructions (1:10,000). Then, the images were captured using confocal microscopy (Zeiss 780/900). The results from comet assays were analyzed quantitatively by using ImageJ software by collectively monitoring more than 30 cells. % DNA in the tail was calculated using the following Eq. [53, 55]:

4 $$\%DNA\, in\, Tail=\left(1-\frac{Total\, head \,DNA \,intensity}{Total \, DNA \, intensity}\right)\times 100\%$$

Neutral electrophoresis solution: TBE (obtained from UNSW store).

Alkaline electrophoresis solution (AES): 200 mM NaOH and 1 mM disodium EDTA pH > 13.

### Mitochondria damage study

JC-1 (5,5’,6,6’-tetrachloro-1,1′3,3’-tetraethyl-imidacarbocyanine iodide, Abcam) was applied to measure membrane potential change of mitochondria. 4T1 cells were cultured in a 96-well plate (Corning) at a density of 5000 cells/well. A 50% confluency, the complete growth media were replaced with media containing different treatments (control (PBS), TME, GQDs (100 µg/ml), GQD (TME)) after 24 h incubation. Then, cells were washed with JC-1 dilution buffer containing 10% FBS and stained with JC-1 working solution (100 µl, 1 µM) in the dark for 15 min. Thereafter, cells were washed with JC-1 dilution buffer twice and then emissions were recorded using Molecular Device SpectraMax iD5 (red: excitation: 530 nm, emission: 590 nm; green: excitation: 475 nm, emission: 530 nm).

## Supplementary Information

**Additional file 1: Table S1.** Band structure and reference equation (1) & (2). **Table S2.** Comparison of Km and Vmax for different catalyst. **Figure S1.** Hydrodynamic size distribution via DLS for GQDs. **Figure S2.** Zeta potential for GQDs. **Figure S3.** FT-IR spectra for GQDs before and after reaction. **Figure S4.** UV-Vis spectrum GQDs (5 μg/ml). **Figure S5.** Relative catalytic activity of GQDs at different pH values (**a**) and different temperature (**b**). **Figure S6.** Normalized absorbance (at 652 nm) of GQDs under TME mimic conditions (TMB (800 μg/ml) + GQDs (100 μg/ml) + 100μM H_2_O_2_) and control groups (MiliQ Water (solvent), TMB (800 μg/ml), TMB (800 μg/ml) + GQDs (100 μg/ml), TMB (800 μg/ml) + 100 μM H_2_O_2_). **Figure S7.** Time course TMB assay - absorbance (at 652 nm) of GQDs (50 μg/ml) with different concentration of H_2_O_2_ (0.1, 0.5, 1, 2 mM). **Figure S8.** Reaction between hydroxyl radical and terephthalic acid (TA). **Figure S9.** Terephthalic acid (TA) intensity over time via fluorescence spectrometry. **Figure S10.** ESR spectra of GQDs (50 μg/ml) + H_2_O_2_ (1 mM) + DMPO (5 mM) comparing with typical hydroxyl radicals pattern with DMPO. **Figure S11.** Tolerance of 4T1 cell line with H_2_O_2_ (0–1 mM) after 24 h. **Figure S12.** Biosafety of GQDs. Cell viability of normal cell NIH-3T3 cells treated with different concentration of GQDs (0–1000 μg/ml) for 24 hr. **Figure S13.** Living cell imaging results: percentage of confluence for 4T1/MCF-7 cell lines under GQDs (CQDs 100 μg/ml) in TME (H_2_O_2_ 100 μM) treatments, control: PBS. Living cell images were recorded on InCyte3. **Figure S14.** Alkaline Comet results, scale bar: 200 μm. **Figure S15.** TEM images for 20 h cellular uptake of GQDs in 4T1 cells.

**Additional file 2.** Time-lapse imaging of 4T1 cells treated with PBS (Control) and GQDs under TME for 24 hours.

**Additional file 3. **Time-lapse imaging of PI-stained 4T1 cells treated with PBS (Control), TME, GQDs (100 μg/ml) and GQDs under TME for 24 hours.

**Additional file 4. **3D cell model -1 cell: 4T1 cells after 24h cellular uptake of GQDs from Z-stack confocal iamges (grey - cell membrane, Dil; Red - nuclei, NucRed647; Blue - GQDs).

**Additional file 5. **3D cell model -4 cells: 4T1 cells after 24h cellular uptake of GQDs from Z-stack confocal images (grey- cell membrane, Dil; Red - nuclei, NucRed647; Blue - GQDs).

## Data Availability

All data in the published article or Additional file 1 are available on request from the authors.
