# Supplementary material for: Understanding of catalytic ROS generation from defect-rich graphene quantum-dots for therapeutic effects in tumor microenvironment
Source: J Nanobiotechnology. 2021 Oct 26;19:340. doi: 10.1186/s12951-021-01053-6 (PMC8547047; doi:10.1186/s12951-021-01053-6)
Supplement: Supplementary file 1 — Additional file 1: Table S1. Band structure and reference equation (1) & (2). Table S2. Comparison of Km and Vmax for different catalyst. Figure S1. Hydrodynamic size distribution via DLS for GQDs. Figure S2. Zeta potential for GQDs. Figure S3. FT-IR spectra for GQDs before and after reaction. Figure S4. UV-Vis spectrum GQDs (5 μg/ml). Figure S5. Relative catalytic activity of GQDs at different pH values (a) and different temperature (b). Figure S6. Normalized absorbance (at 652 nm) of GQDs under TME mimic conditions (TMB (800 μg/ml) + GQDs (100 μg/ml) + 100μM H2O2) and control groups (MiliQ Water (solvent), TMB (800 μg/ml), TMB (800 μg/ml) + GQDs (100 μg/ml), TMB (800 μg/ml) + 100 μM H2O2). Figure S7. Time course TMB assay - absorbance (at 652 nm) of GQDs (50 μg/ml) with different concentration of H2O2 (0.1, 0.5, 1, 2 mM). Figure S8. Reaction between hydroxyl radical and terephthalic acid (TA). Figure S9. Terephthalic acid (TA) intensity over time via fluorescence spectrometry. Figure S10. ESR spectra of GQDs (50 μg/ml) + H2O2 (1 mM) + DMPO (5 mM) comparing with typical hydroxyl radicals pattern with DMPO. Figure S11. Tolerance of 4T1 cell line with H2O2 (0–1 mM) after 24 h. Figure S12. Biosafety of GQDs. Cell viability of normal cell NIH-3T3 cells treated with different concentration of GQDs (0–1000 μg/ml) for 24 hr. Figure S13. Living cell imaging results: percentage of confluence for 4T1/MCF-7 cell lines under GQDs (CQDs 100 μg/ml) in TME (H2O2 100 μM) treatments, control: PBS. Living cell images were recorded on InCyte3. Figure S14. Alkaline Comet results, scale bar: 200 μm. Figure S15. TEM images for 20 h cellular uptake of GQDs in 4T1 cells. [file 12951_2021_1053_MOESM1_ESM.pdf]

Supporting information

Understanding of catalytic ROS generation from defect-rich graphene quantum-dots for therapeutic effects in tumor microenvironment

Xichu Wang, Chuangang Hu, Zi Gu*, Liming Dai*

Australian Carbon Materials Centre (A-CMC), School of Chemical Engineering, University of New South Wales, Sydney, NSW,2052, Australia.

Supplementary Table S1 Band structure and reference equation (1) & (2)

Valence band (eV)	Conduction band(eV)	Band gap (eV)
-6.26	-4.47	1.77

$$E_g = \frac{1242}{\lambda_{onset}} \quad (1)[1]$$

$$E(HOMO) = -e[E_{OX}^{onset} + 4.4] \quad (2)[1]$$

Supplementary Table S2 Comparison of Km and Vmax for different catalyst

Catalyst	Km(mM)	Vmax (x10^{-8} M/s)
GQDs	0.86	5.4
Horseradish peroxidase (HRP)[2]	3.7	8.71
Fe3O4 MNPs [2]	154	9.78
MOF Fe-MIL-88NH2[3]	2.06	2.06
N-PCNSs[4]	130	32.5

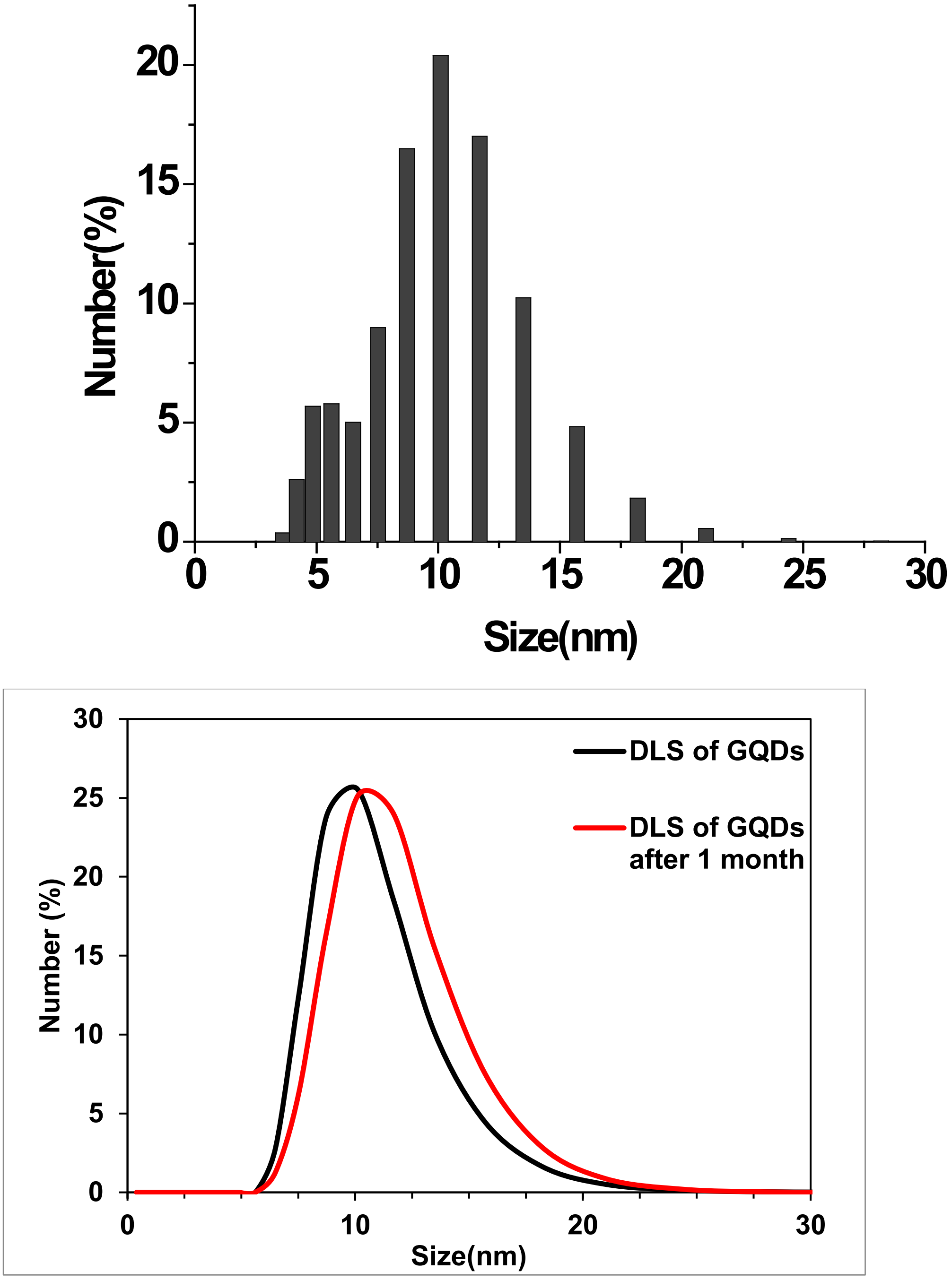

Supplementary Figure S 1 Hydrodynamic size distribution of GQDs via DLS.

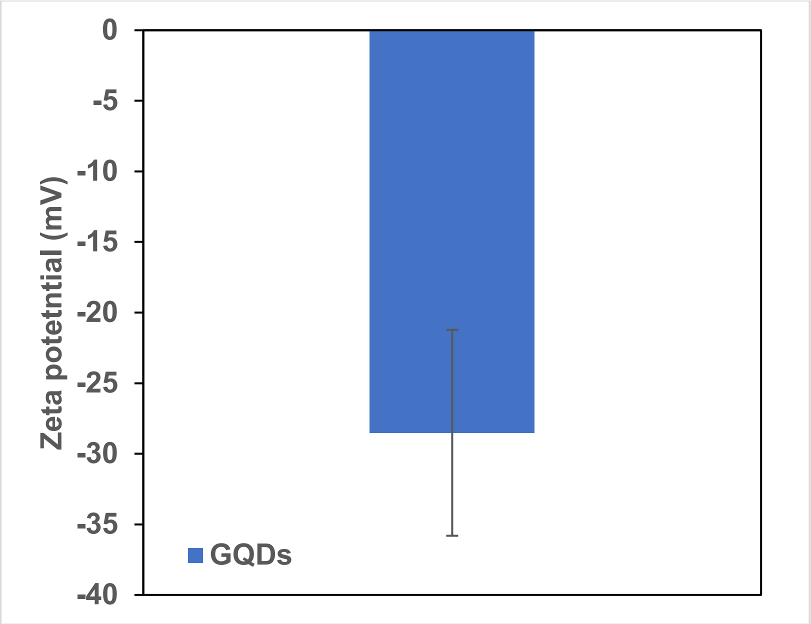

Supplementary Figure S 2 Zeta potential of GQDs.

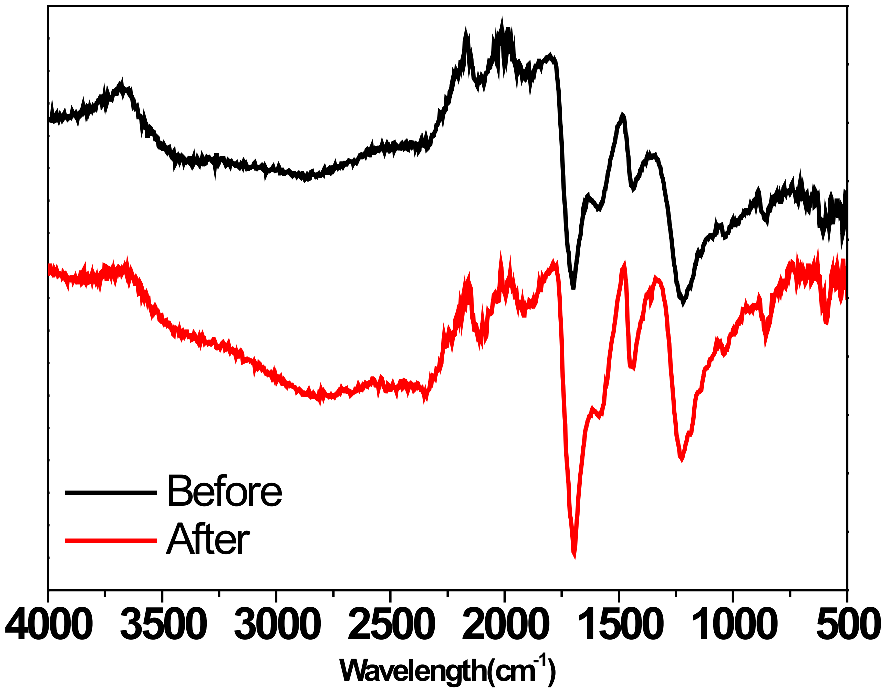

Supplementary Figure S 3 FT-IR spectra for GQDs before and after reaction.

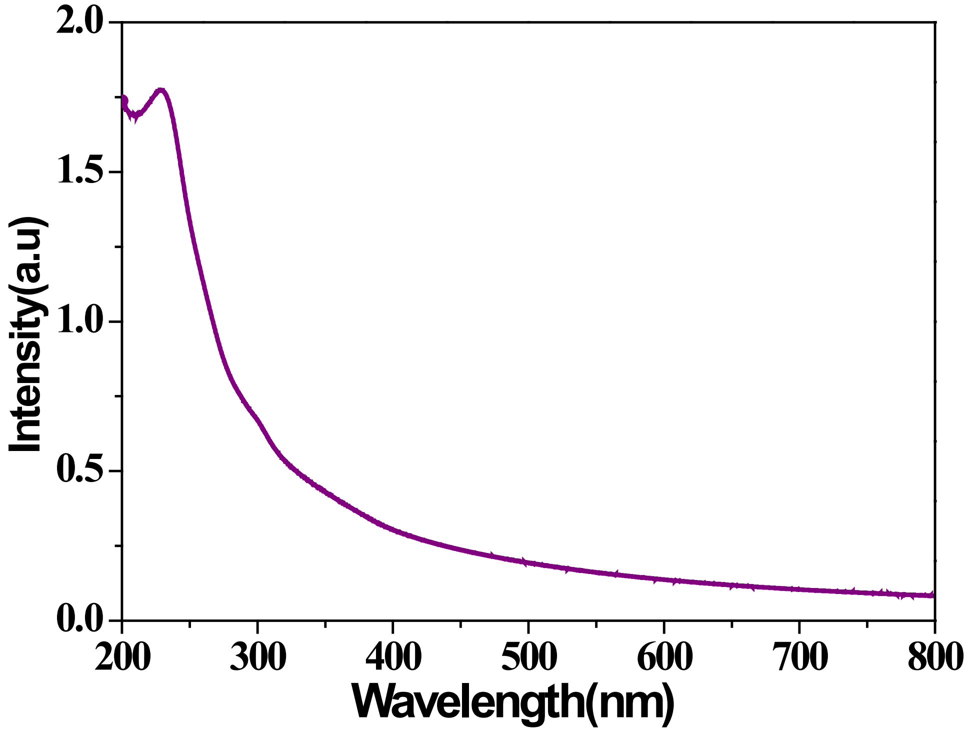

Supplementary Figure S 4 UV-Vis spectrum GQDs (5 µg/ml).

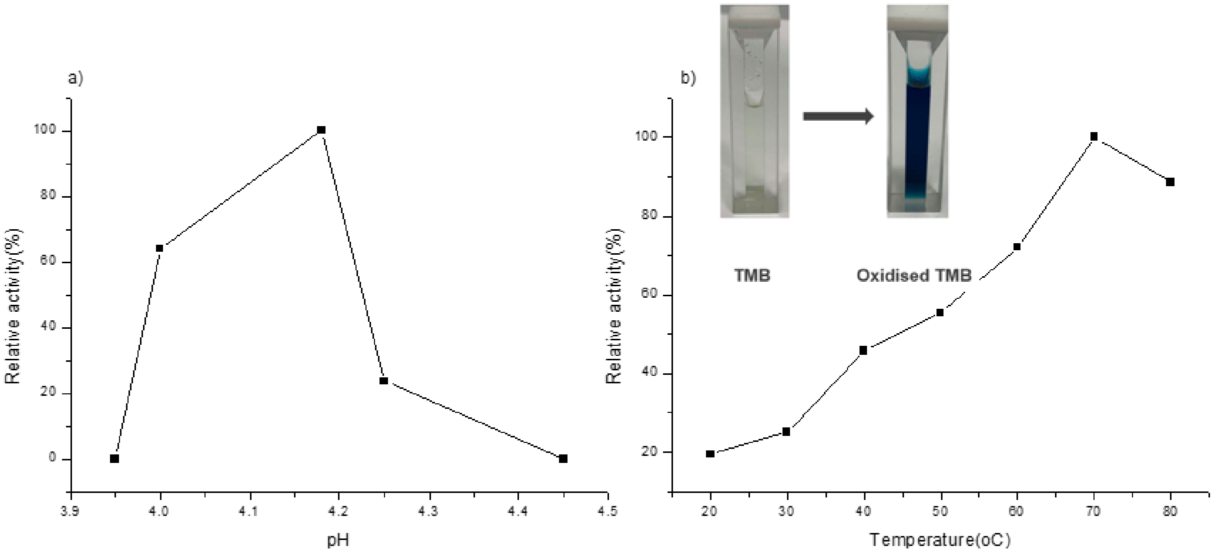

Supplementary Figure S 5 Relative catalytic activity of GQDs at different pH values (a) and different temperature (b).

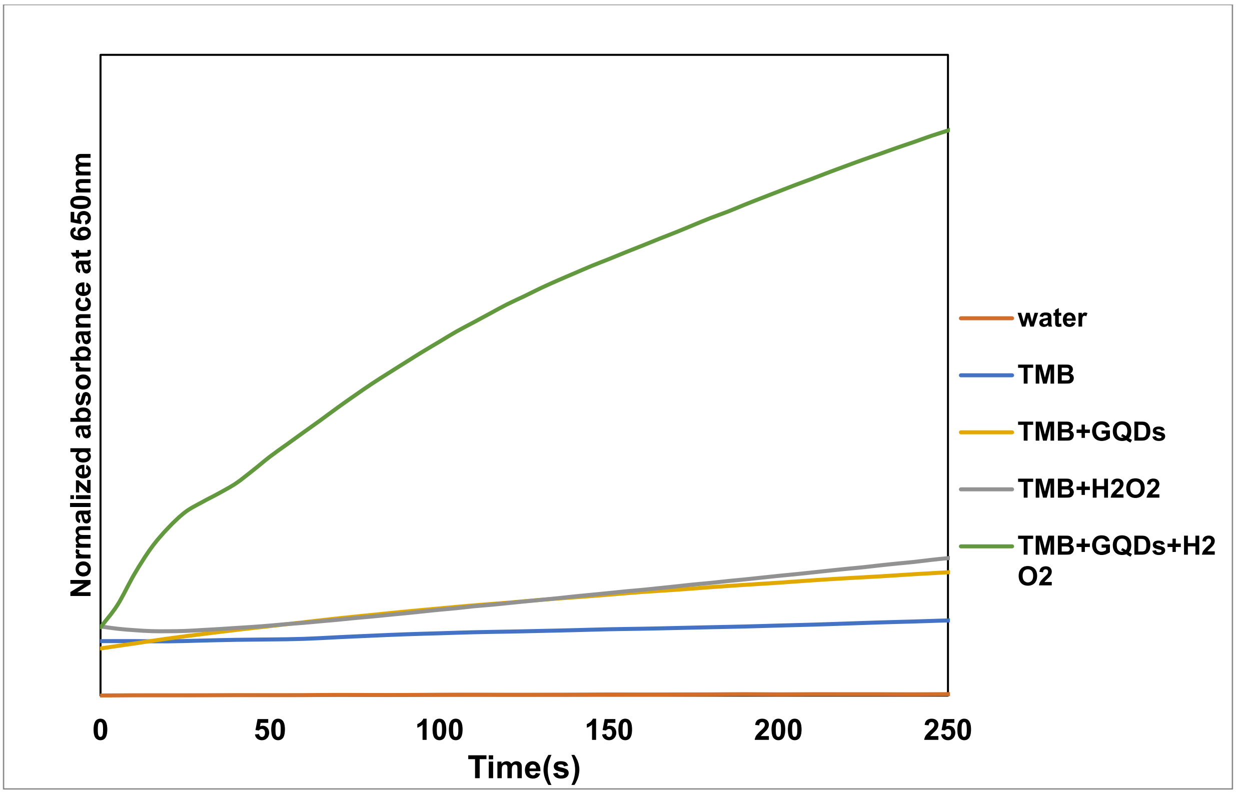

Supplementary Figure S 6 Normalized absorbance (at 652nm) of GQDs under TME mimic conditions (TMB (800µg/ml) +GQDs (100µg/ml)+100µM H_2O_2) and control groups (MiliQ Water (solvent), TMB (800µg/ml), TMB (800µg/ml) +GQDs (100µg/ml), TMB (800µg/ml) + 100µM H_2O_2).

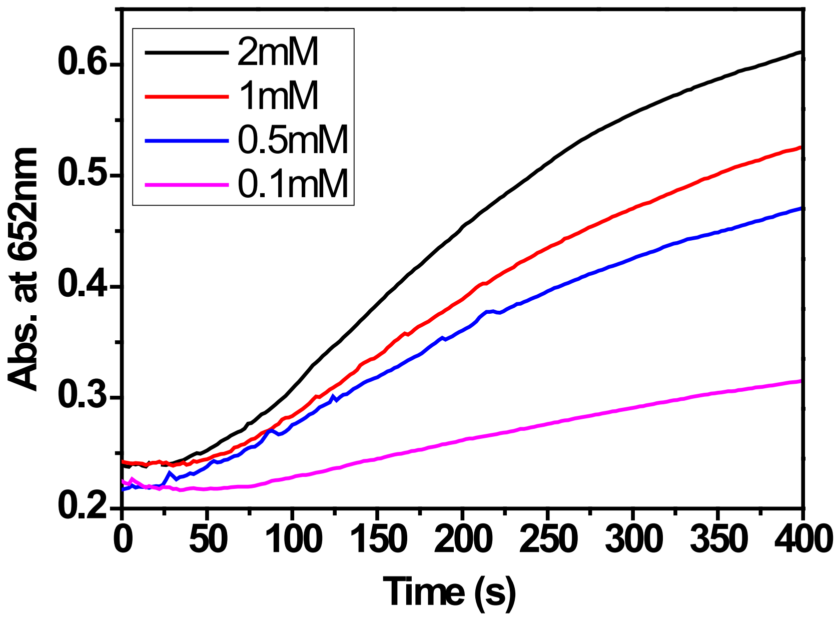

Supplementary Figure S 7 Time course TMB assay - absorbance (at 652nm) of GQDs (50 µg/ml) with different concentration of H_2O_2(0.1,0.5,1,2mM)

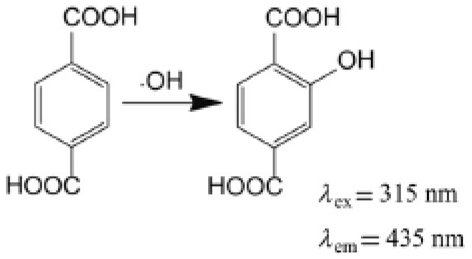

Supplementary Figure S 8 Reaction between hydroxyl radical and terephthalic acid (TA),CQDs(GQDs) Fluorescence spectra of GQDs in PBS . [5]

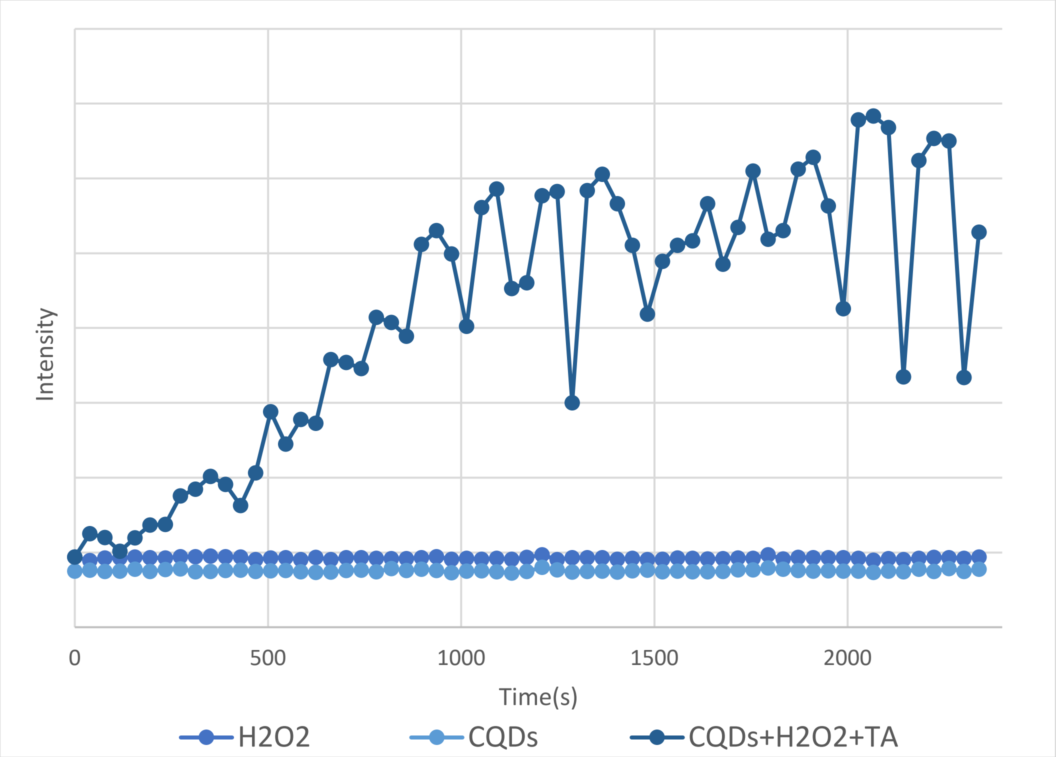

Supplementary Figure S 9 Terephthalic acid (TA) intensity over time via flurescence spectrometry.

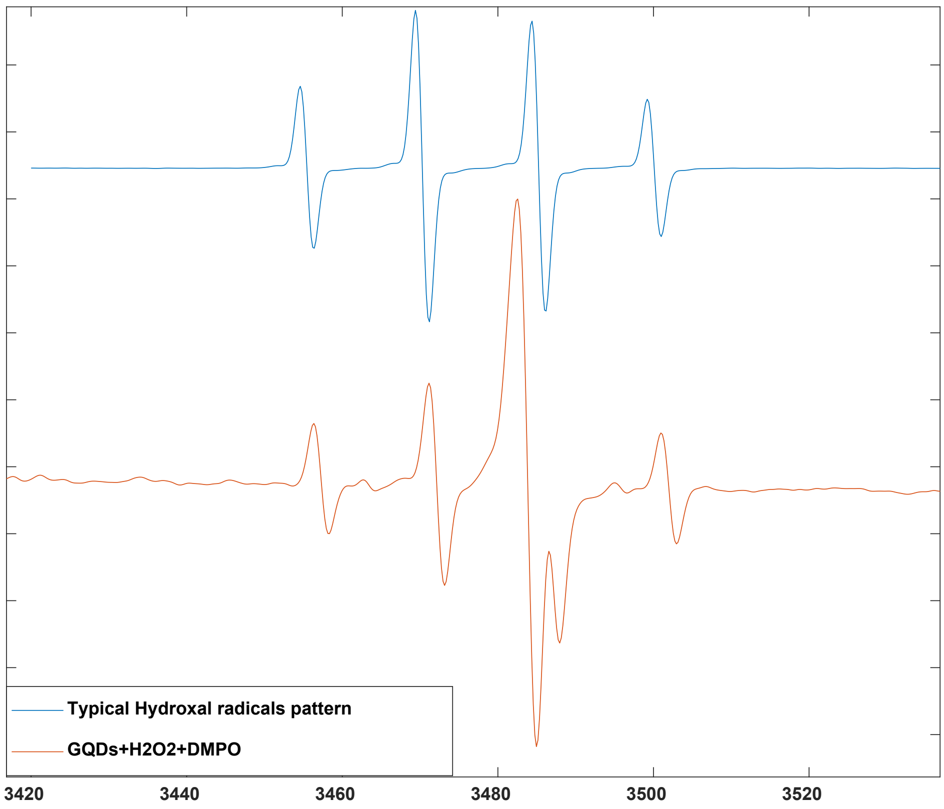

Supplementary Figure S 10 ESR spectra of GQDs(50 µg/ml) +H_2O_2 (1mM) +DMPO (5 mM) (comparing with typical hydroxal radicals pattern with DMPO.

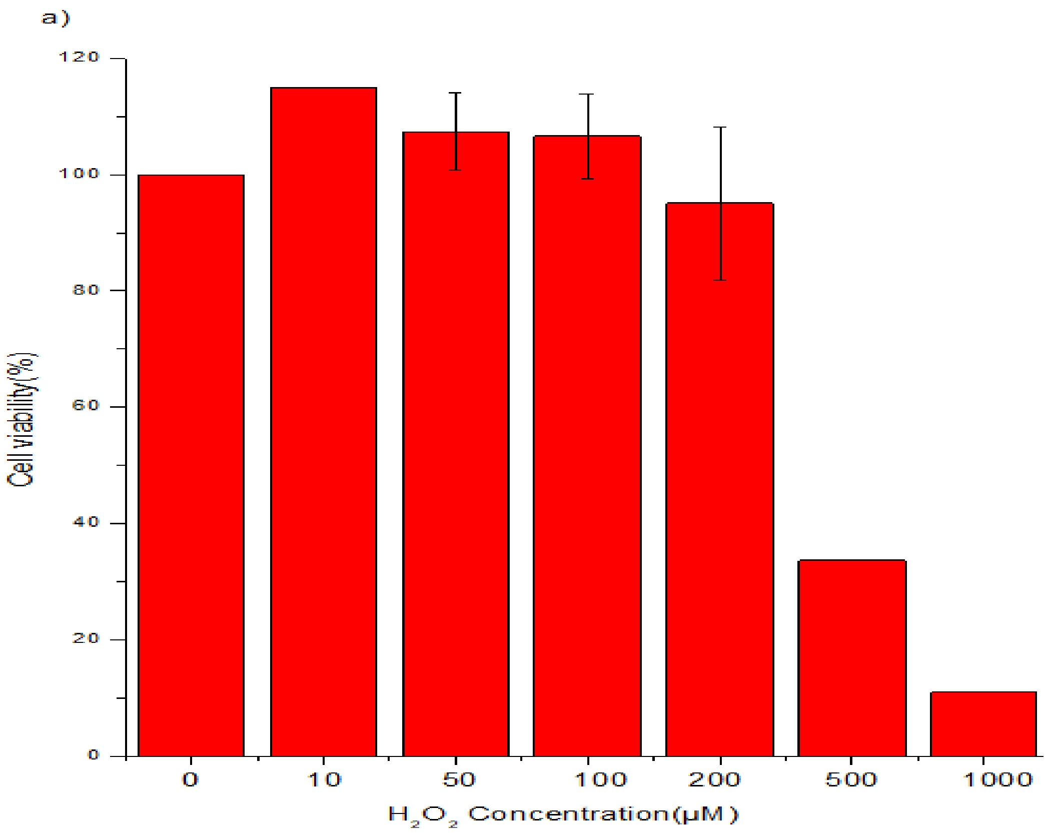

Supplementary Figure S 11 Tolerance of 4T1 cell line with H_2O_2(0-1mM) after 24h.

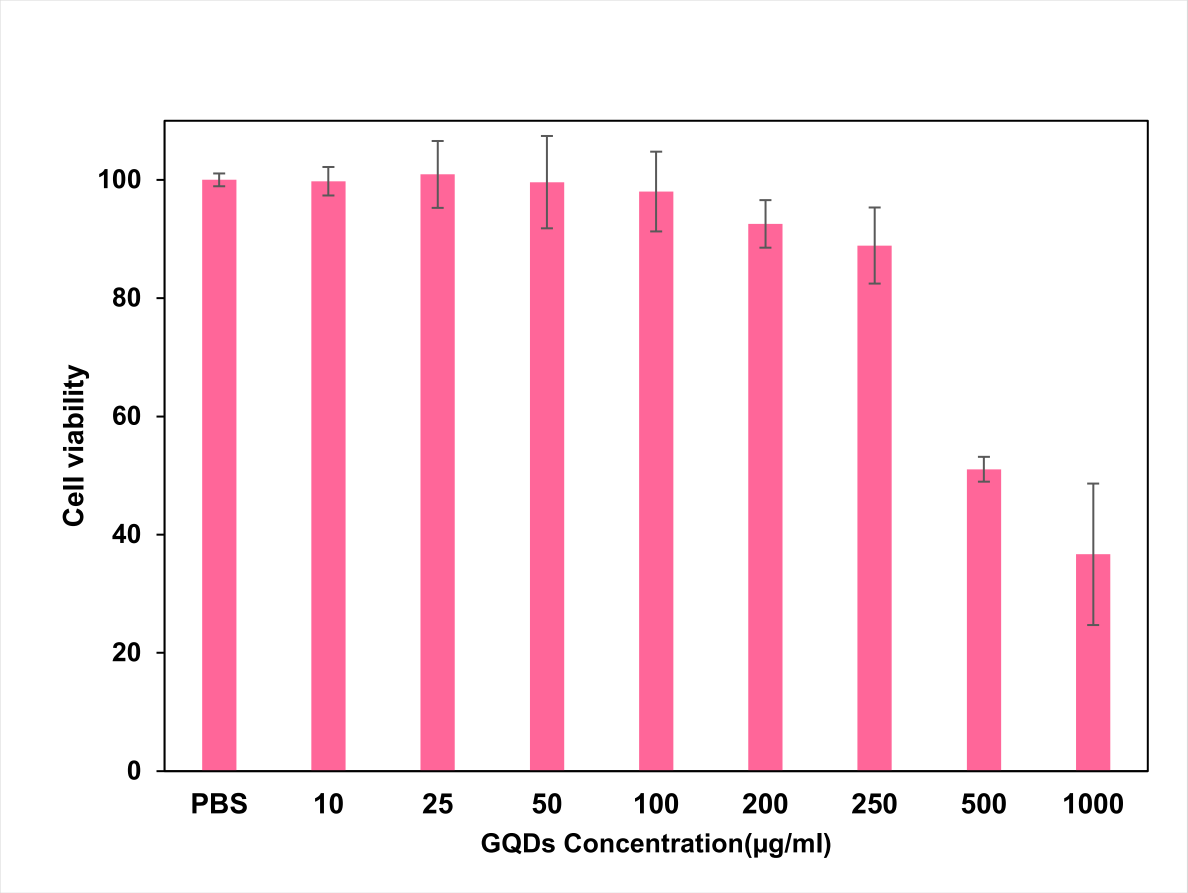

Supplementary Figure S 12 Biosafety of GQDs. Cell viability of normal cell NIH-3T3 cells treated with different concentration of GQDs (0-1000 µg/ml) for 24hr.

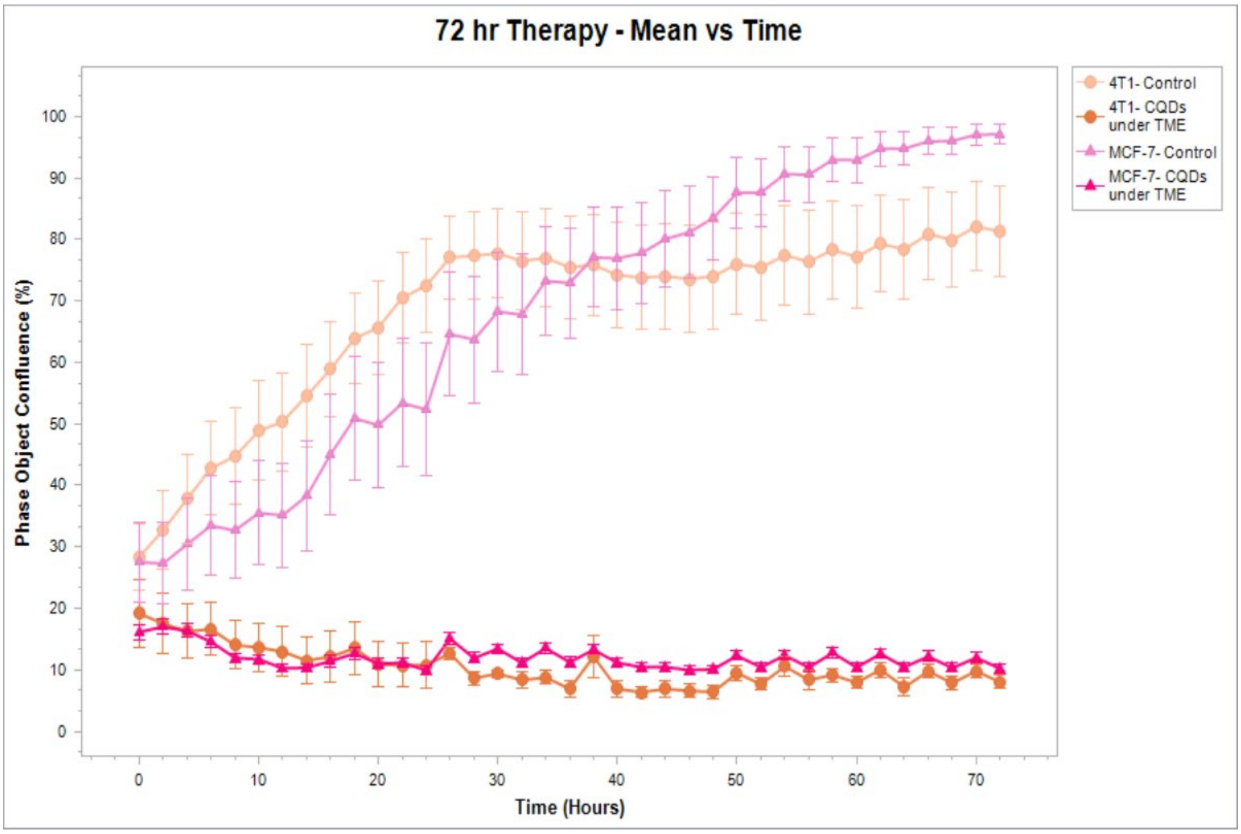

Supplementary Figure S 13 Living cell imaging results: percentage of confluence for 4T1/MCF-7 cell lines under GQDs (CQDs 100 µg/ml) in TME (H_2O_2 100µM) treatments, control: PBS. Living cell images were recorded on InCyte3.

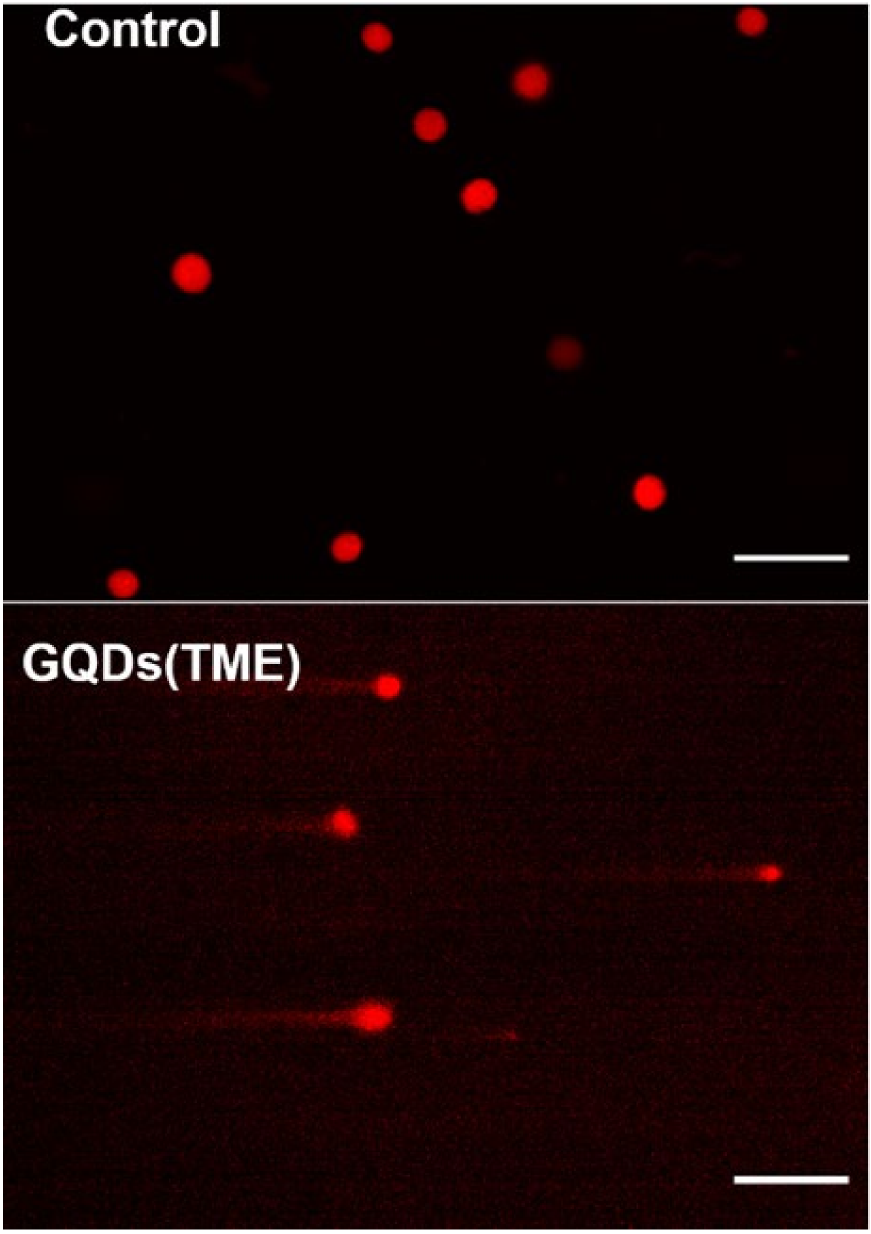

Supplementary Figure S 14 Alkaline Comet results, scale bar:200µm.

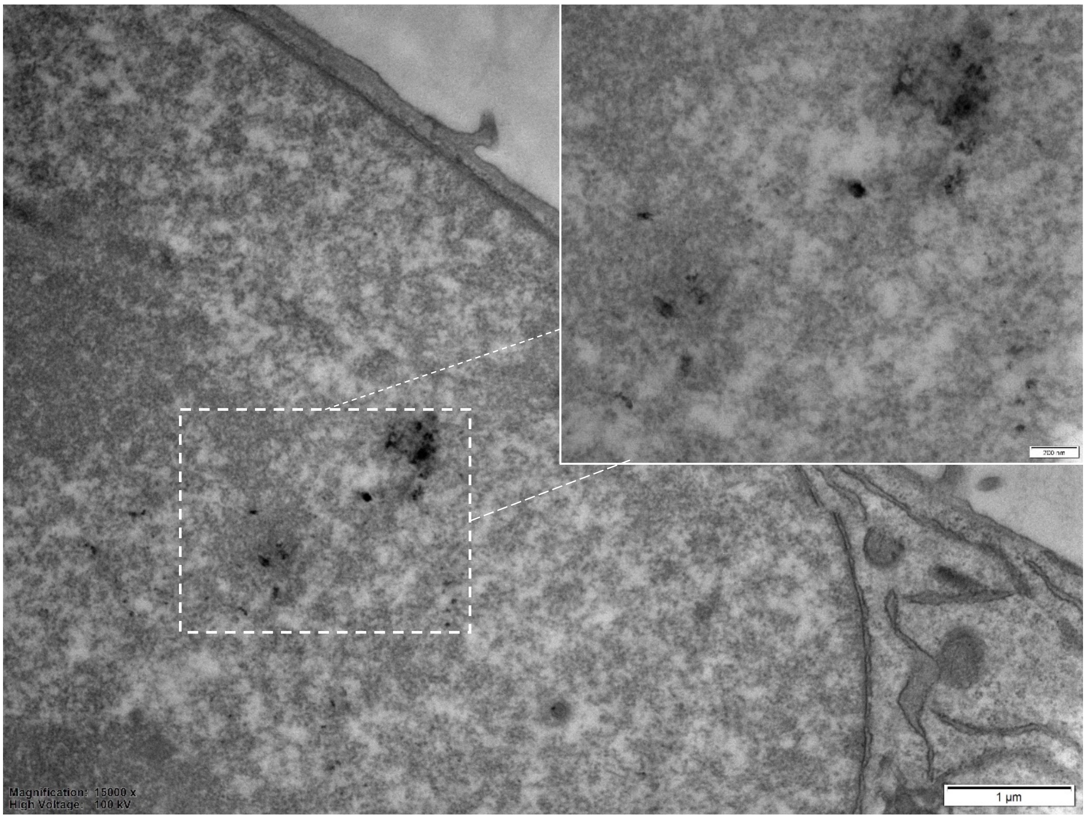

Supplementary Figure S 15 TEM images for 20-h cellular uptake of GQDs in 4T1 cells.

References

1. Leonat, L., Sbârcea, G. & Brańzoi, I. V. Cyclic voltammetry for energy levels estimation of organic materials. *UPB Sci. Bull. Ser. B Chem. Mater. Sci.* **75**, 111–118 (2013).
2. Gao, L. *et al.* Intrinsic peroxidase-like activity of ferromagnetic nanoparticles. *Nat. Nanotechnol.* **2**, 577–583 (2007).
3. Liu, Y. L., Zhao, X. J., Yang, X. X. & Li, Y. F. A nanosized metal–organic framework of Fe-MIL-88NH2 as a novel peroxidase mimic used for colorimetric detection of glucose. *Analyst* **138**, 4526–4531 (2013).
4. Fan, K. *et al.* In vivo guiding nitrogen-doped carbon nanozyme for tumor catalytic therapy. *Nat. Commun.* **9**, (2018).
5. Sun, H., Gao, N., Dong, K., Ren, J. & Qu, X. Graphene quantum dots-band-aids used for wound disinfection. *ACS Nano* **8**, 6202–6210 (2014).
