# Supplementary figures and images for: Understanding of catalytic ROS generation from defect-rich graphene quantum-dots for therapeutic effects in tumor microenvironment
Source: J Nanobiotechnology. 2021 Oct 26;19:340. doi: 10.1186/s12951-021-01053-6 (PMC8547047; doi:10.1186/s12951-021-01053-6)

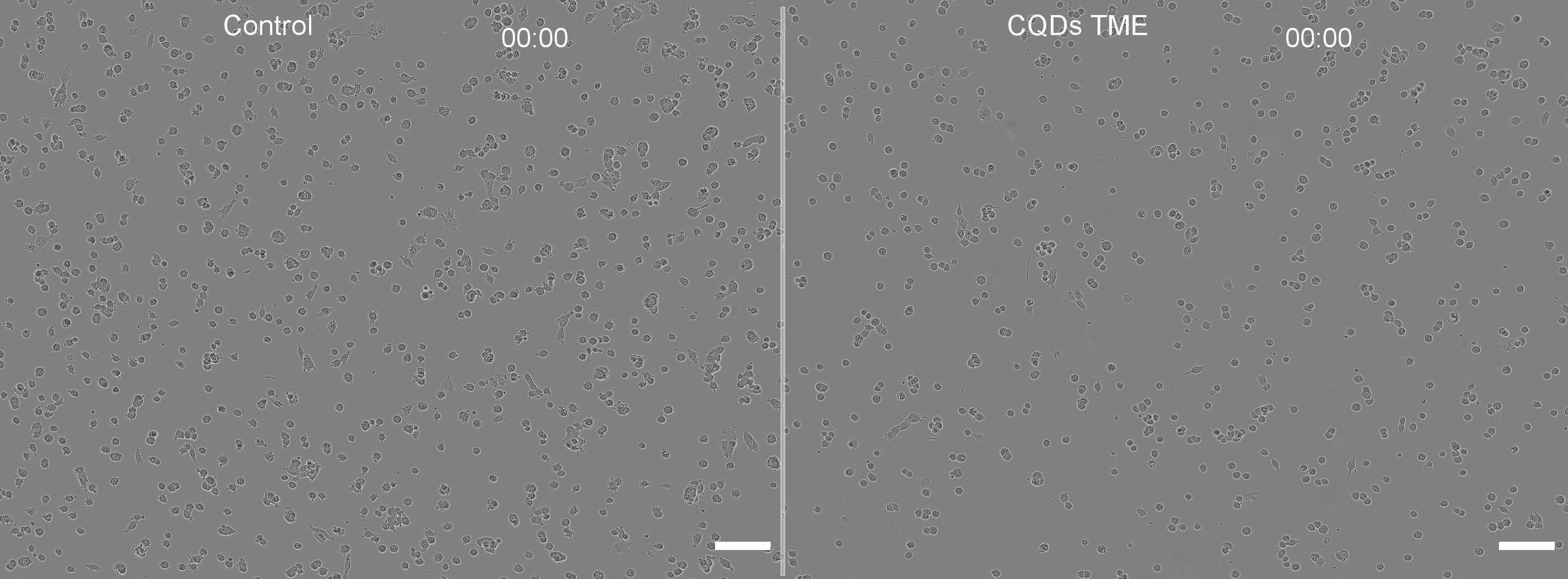

Supplement: Supplementary file 2 — Additional file 2. Time-lapse imaging of 4T1 cells treated with PBS (Control) and GQDs under TME for 24 hours. [file 12951_2021_1053_MOESM2_ESM.gif]

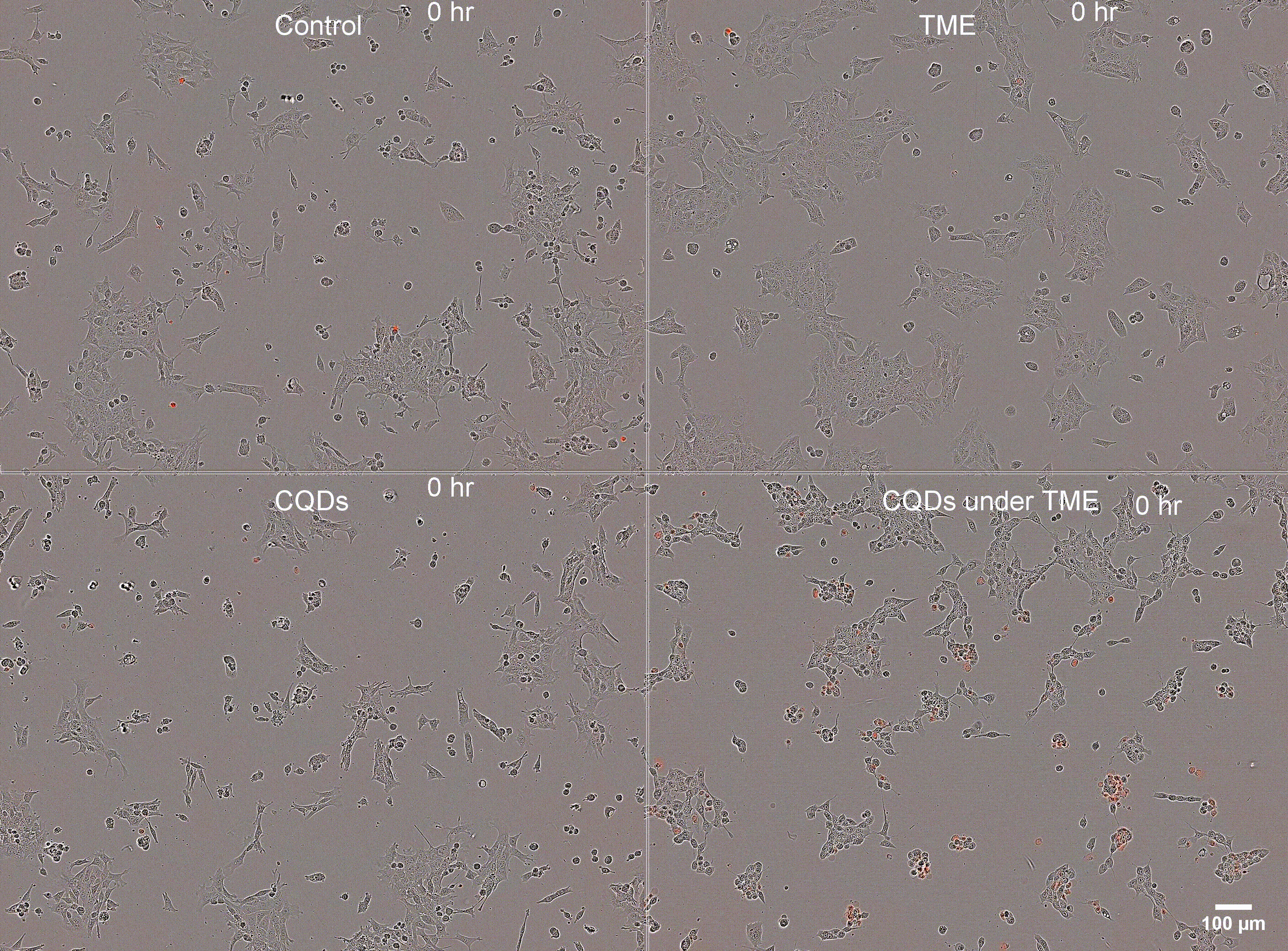

Supplement: Supplementary file 3 — Additional file 3. Time-lapse imaging of PI-stained 4T1 cells treated with PBS (Control), TME, GQDs (100 μg/ml) and GQDs under TME for 24 hours. [file 12951_2021_1053_MOESM3_ESM.gif]

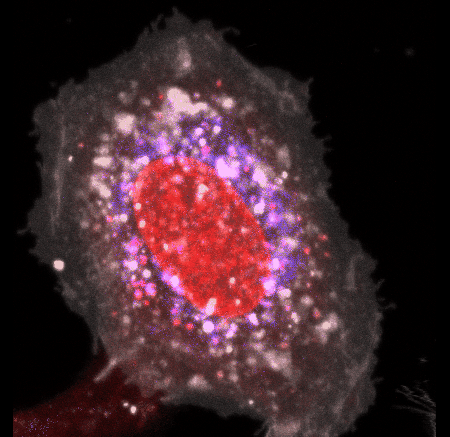

Supplement: Supplementary file 4 — Additional file 4. 3D cell model -1 cell: 4T1 cells after 24h cellular uptake of GQDs from Z-stack confocal iamges (grey - cell membrane, Dil; Red - nuclei, NucRed647; Blue - GQDs). [file 12951_2021_1053_MOESM4_ESM.gif]

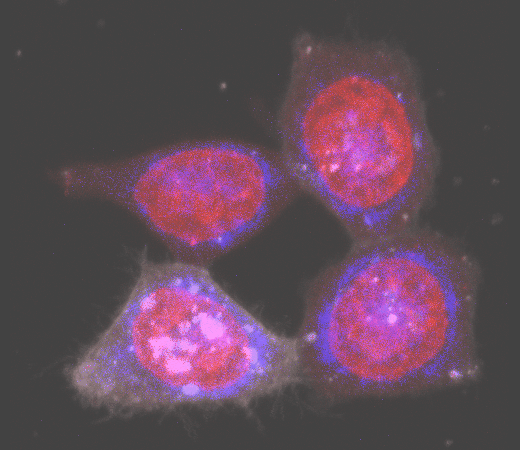

Supplement: Supplementary file 5 — Additional file 5. 3D cell model -4 cells: 4T1 cells after 24h cellular uptake of GQDs from Z-stack confocal images (grey- cell membrane, Dil; Red - nuclei, NucRed647; Blue - GQDs). [file 12951_2021_1053_MOESM5_ESM.gif]
